# Mutation rate analysis via parent–progeny sequencing of the perennial peach. II. No evidence for recombination-associated mutation

**DOI:** 10.1098/rspb.2016.1785

**Published:** 2016-10-26

**Authors:** Long Wang, Yanchun Zhang, Chao Qin, Dacheng Tian, Sihai Yang, Laurence D. Hurst

**Affiliations:** 1 State Key Laboratory of Pharmaceutical Biotechnology, School of Life Sciences, Nanjing University, Nanjing 210023, People's Republic of China; 2 The Milner Centre for Evolution, Department of Biology and Biochemistry, University of Bath, Bath BA2 7AY, UK

**Keywords:** peach, mutation rate, crossover rate, domestication

## Abstract

Mutation rates and recombination rates vary between species and between regions within a genome. What are the determinants of these forms of variation? Prior evidence has suggested that the recombination might be mutagenic with an excess of new mutations in the vicinity of recombination break points. As it is conjectured that domesticated taxa have higher recombination rates than wild ones, we expect domesticated taxa to have raised mutation rates. Here, we use parent–offspring sequencing in domesticated and wild peach to ask (i) whether recombination is mutagenic, and (ii) whether domesticated peach has a higher recombination rate than wild peach. We find no evidence that domesticated peach has an increased recombination rate, nor an increased mutation rate near recombination events. If recombination is mutagenic in this taxa, the effect is too weak to be detected by our analysis. While an absence of recombination-associated mutation might explain an absence of a recombination–heterozygozity correlation in peach, we caution against such an interpretation.

## 1. Introduction

Both mutation rates and recombination rates vary between species and between regions within a genome [1,2]. In the accompanying paper, we ask, via parent–progeny sequencing of the peach, whether woody perennials might have low mutation rates [3–5] compared with fast-growing annuals and whether hybrid strains have higher mutation rates [6]. Here, employing the same data, we focus on the possibilities that recombination might be mutagenic [7,8] and whether the recombination rate of domesticated peach is higher than that of wild peach, there commonly being a suggestion that domestication is associated with raised recombination rates [9–11]. If both are true then some variation between genomic regions and between strains in the mutation rate may be attributable to recombination-associated mutation.

The idea that recombination, or meiosis more generally, might be mutagenic stems from the work of Magni [7,8] in which he observed a higher mutation rate in meiotic than mitotic yeasts. From a mechanistic view, a correlation could be expected between mutations raised from double-strand break (DSB)-repairing errors and those DSBs occurring in homologous recombination [12]. If recombination is mutagenic, then we expect domains of high recombination to be domains of high rates of new mutations. The hypothesis has proven highly controversial, with indirect evidence both consistent [13–16] and inconsistent [17–20] with the hypothesis. The best indirect data, however, argue against the possibility. Notably, in 1000 Genomes data there is no increased variation around recombinogenic hotspot motifs [21]. Moreover, evidence from a correlation between recombination and the rate of putatively neutral evolution [13,14,16], now appears to be better explained as a consequence of biased gene conversion [22]. While then, until recently, convincing direct evidence for recombination being mutagenic has been lacking (for review, see [23]), even more recent direct evidence in humans [24], yeast [25] and bees [1] supports the hypothesis that recombination is mutagenic, although the effect might be very weak. Were recombination mutagenic, we might also predict that species with higher recombination rates should have a higher mutational input. However, higher divergence might in turn lead to reduced recombination rates [20] making prediction harder. There are numerous alternative suggested determinants of intragenomic variation in the mutation rate: for example, it correlates with local sequence context [26], including presence of insertion/deletions (indels) [27], replication timing [28], as well as possibly epigenetic effects such as chromatin organization [29].

The parent–progeny sequencing data that enables us to estimate the mutation rate, also enables us to determine the recombinational landscape of peach. Domesticated species are conjectured to have been indirectly selected for high recombination rates [9–11]. This is because directional selection owing to domestication, might select for modifier alleles that increase the recombination rate; either because drift permits build-up of linkage disequilibrium (especially in smaller populations) or because epistatic effects generate linkage disequilibria among selected loci [11]. Evidence of increased recombination in domesticated plant species, based on the analysis of chiasmata number, is supportive of such a link [30]. A correlation between domestication and high recombination rate could be owing to high recombination prior to domestication, as a form of preadaptation to domestication [31], but current evidence argues against this [30]. However, more recent sequence data-based estimates of recombination rates in mammals contradict the domestication–recombination hypothesis [32]. It is unclear whether this difference in results between analyses reflects a taxonomic (plant–mammal) or methodological (chiasmata counts versus direct recombination inference) difference. Here then we ask whether domesticated peach has a higher recombination rate than a wild close relative and whether mutations occur more often near recombination break points.

## 2. Material and methods

We constructed three parent–progeny groups (groups I–III). Each group has an F_1_ parent tree together with its selfed F_2_ progeny. Groups I and II are low heterozygosity intraspecific crosses employing young (group I) and old (group II) F_1_s, while group III F_1_ is an interspecific cross. Group I included one F_1_ (*Prunus persica*) and 24 selfed F_2_ samples (144F2-1 to -24 in the electronic supplementary material, table S1). Group II included one weakly heterozygous F_1_ (*Prunus mira*, a wild peach) and nine selfed F_2_ samples (GZTH-S1 to –S5, –S7 to –S9 and GZTH-5). The interspecific crossing group (group III) included four ancestral parents, one heterozygous F_1_ (*Prunus davidiana* × *P. persica*) and 30 F_1_ selfed F_2_ samples (NE1–NE30 in the electronic supplementary material, table S1). In total, 70 peach samples, including four ancestral parents from group III, three F_1_ parents (i.e. each group with one F_1_ sample) and 63 F_2_s were selected for whole-genome resequencing. This was done with high sequence quality (base quality Q20 ≥ 95%), high depth (51.3× on average and ranging from 38.3× to 65.8×) and relatively long reads (150 bp × 2, paired end sequencing strategy by Hiseq4000 platform; electronic supplementary material, table S1).

For further methods pertinent to sampling, sequencing and alignment, variant calling, de novo mutation identification, Sanger validation of mutation calls, estimation of mutation rate and estimation of heterozygosity, we refer the reader to the prior paper. A full methodology pertinent for both papers is also presented as the electronic supplementary material.

### (a) Variant calling and marker identification

Raw variants for each sample were called using GATK HaplotypeCaller (HC) in GVCF mode [33]. For recombination analysis, markers with low confidence could hamper the identification of true recombinant blocks; therefore, it is important to exclude false variant calls as thoroughly as possible. To generate a high-confidence variant set, we only use bi-allelic variant loci with: (i) quality greater than or equal to 50; (ii) a depth no less than 10 and not exceeding 80; and (iii) more than half of samples contain informative calls in each group. To reduce the genotyping errors, we also required a reference allelic ratio of 0–5% or 95–100% to be considered as a confident homozygote, while 30–70% was required to make a confident heterozygous call. A confident marker was thus identified where the F_1_ samples were present in a confident heterozygous status. This allele-balance filter is efficient for removing genotyping errors owing to sequencing errors or possible contaminates, as those errors were most likely at a low frequency. However, mapping errors owing to highly similar paralogous sequences could also result in pseudo-heterozygosity. To minimize these errors, we remove those markers residing in large structural variant (SV) regions of F_1_ samples compared with the reference genome in each group. The SVs were detected by combining three different algorithms: a read-depth approach (CNVnator) [34], a split-read approach (Pindel) [35] and from the analysis of discordant pairs (Breakdancer) [36]. CNVnator (v. 0.3) was run with a bin size of 100 bp, which predicts large deletions and duplications. Pindel (v. 0.2.5b6) was run with default options. Results were collected for large deletions (greater than or equal to 100 bp), inversions and translocations. Deletion, duplication and inversion results were also collected from Breakdancer (v. 1.1.2) with default settings. We generated a union set of results collected from all three approaches without further filtering. SVs with a size smaller than 100 kbp were directly used. We also include 200 bp flanking regions of all inversion events. For SVs larger than 100 kbp, we use the 400 bp flanking regions around each predicted SV breakpoint.

### (b) Detection of crossover events

For interspecific F_2_ samples, we first genotyped each marker as *P. persica*-homozygous, *P. davidiana*-homozygous or heterozygous, by comparing against these parents. The markers were then clustered using a ‘seeding and extension’ approach to form the original inherited blocks. First, fragments with 25 consecutive markers of the same genotype and a length over 10 kbp were chosen as a seed; adjacent seeds with same genotype were then merged into larger fragments (blocks) until all adjacent fragments were of different genotypes. Each block was further extended to the furthest marker of the same genotype where the overall proportion of this genotype started to decline. This algorithm has been implemented in the script ‘vcf_process.pl’ and is available from https://github.com/wl13/BioScripts. Finally, all boundaries of blocks were manually inspected and revised. The location of crossover (CO) events was determined as the location where block genotype switched.

For the intraspecific *P. persica* group, it is difficult to first genotype each marker as neither of the parental individuals were available. Thus, we only genotyped those markers as homozygous or heterozygous at first, and formed the blocks using the same clustering method mentioned earlier. This rests on the assumption of there being only a negligible chance for two CO events to be observed in a very narrow region (i.e. within two adjacent markers) from a single F_2_ genome. This is reasonable as the two haplotypes of the same F_2_ genome came from independent meiotic processes. Once the initial blocks were formed, the F_1_ and other F_2_ chromosomes could then be phased according to those homozygous blocks (electronic supplementary material, figure S1*a*). For each chromosome, we picked out a sample in which only a homozygous genotype was observed. As the selected sample consists of two identical haplotypes (defined as ‘Haplotype1’), the F_1_ chromosome as well as other F_2_ chromosomes could thus be phased through comparison with this haplotype (electronic supplementary material, figure S1*b*). This process also relaxed the previous assumption and was robust to possible phasing errors (electronic supplementary material, figure S1*c*). The final phased blocks were used to detect CO events as described before.

In order to make sure the stringent filtering steps did not remove many true variants and lead to an underestimation of CO events, we also identified inherited blocks and CO events before each filtering step was implemented. Through comparison of the CO events identified in those intermediate steps with the final results, we identified those filtered CO events that were always shared among many different individuals, which was not likely to happen in the randomly sampled F_2_ samples. Manual inspection of those regions also confirmed the non-proper mapping status and artefactual clustering of markers (standard error of distances between each two adjacent markers more than 100) in those regions.

The *P. mira* F_1_ individual was estimated to have a slightly higher heterozygosity (0.0029) than *P. persica* F_1_ cross (0.0027); however, the mapping results of the *P. mira* group were largely subjected to the genome rearrangements observed between *P. mira* and *P. persica*. Given a rough estimation, about half of the covered regions were associated with abnormal depth, non-proper insert size or orientation, which was even higher than estimated for *P. davidiana*. The large-scale genomic rearrangement between *P. mira* and *P. persica* made the results less reliable as regards the CO results for *P. mira* group II. Furthermore, group II had a relative small size of samples compared with the other two groups, which also hindered a solid conclusion derived from this group. Therefore, we did not include the detailed CO results of this group (II) in the current study, and only gave a conservative estimate of its lower boundary by removing the most ambiguous results through manual inspection.

### (c) Statistical analysis

The CO coldspot and hotspot regions were detected by first dividing the whole genome in non-overlapping 500 kbp windows. Midpoints of CO breaks were used as the location of CO events and were counted for each window. Windows with similar CO numbers were merged. All windows after merging were tested using a Monte Carlo process, with 10 000 randomizations of shuffling all CO events across the whole genome to derive the *p*-values. Regions with observed CO events significantly deviating (*p* < 0.05) from the expectation of randomizations were defined as hotspot regions (more than expectation) or coldspot regions (less than expectation), respectively.

To test whether the CO rate was correlated with the mutation rate, we binned the genome into 500 kbp, 1 Mbp, 2 Mbp and 5 Mbp domains. CO events and mutations were collected from both intraspecific *P. persica* group I and interspecific group III. Bins overlapping peri-centromeric regions were discarded due to recombination suppression in those regions. The relationship was tested using Spearman's rank correlation.

To further test whether the CO rate was correlated with the intraspecific population diversity, 70 *P. persica* individuals were collected from published data [37]. All reads were mapped to the reference genome using BWA-backtrack algorithms [38], followed by marking of PCR duplicates (i.e. probably PCR amplification artefacts) and realignment processes as described before. Both variants and non-variant sites were called with HC in GVCF mode. Variant sites with more than half missing alleles or with a non-reference allele frequency of less than 7 (e.g. 5% of all 70 diploid individuals) were excluded to reduce false positive calls.

The population diversity was calculated as the average pairwise differences among all possible pairs. The pairwise difference was defined as the per site nucleotide difference between each of the two compared individuals, e.g. 1 would be counted for a difference between two different homozygous genotypes while 0.5 would be counted for a difference between a homozygous genotype and a heterozygous genotype. The pairwise differences were obtained by first summing up all nucleotide differences in a window, then dividing by the number of informative sites (sites genotyped in both individuals) in the same window. For each pair, only windows with more than 50% informative sites were considered as an informative pair in this window. Windows with less than 1208 informative pairs (e.g. 50% of all total 2415 pairs) were discarded from the correlation test. Statistics and correlation tests were performed in R [39].

## 3. Results

### (a) Identification of accurate markers in each parent–progeny peach group

To ensure the accuracy of the called markers used in recombination analysis in each parent–progeny group, several strategies were employed (see Material and methods for details). In total, 302 164, 132 572 and 1 110 854 reliable single nucleotide polymorphisms, as well as 37 856, 21 426 and 115 874 small insertion/deletion (indel) markers were called for groups I, II and III, respectively. This corresponds to an average of 1.51, 0.68 and 5.44 variant markers per kilo base pair for groups I, II and III, respectively. These markers were used to identify the genotypes of heterozygous or homozygous regions in these F_2_ genomes. In our three parent–progeny groups, the average nucleotide diversity (number of nucleotide differences per site) were approximately 0.29%, 0.27% and 1.24% at the whole-genome level between the two haplotypes derived from a single F_1_ in group I, II and III, respectively. As expected, an approximate 4.4-fold higher diversity was detected in the interspecific crossing group compared with the intraspecific groups.

### (b) The recombination rate is consistent with low rates in woody perennials

Before addressing the question of whether peach has high recombination rates compared with wild relatives and whether mutation and recombination are coupled, we first sought to determine aspects of the basic biology of recombination in peach. For example, for benchmarking, we ask whether our rate estimation is consistent with prior estimates [40,41] and with the suggestion that woody perennials have overall low rates [5,42,43].

**Figure 1.**
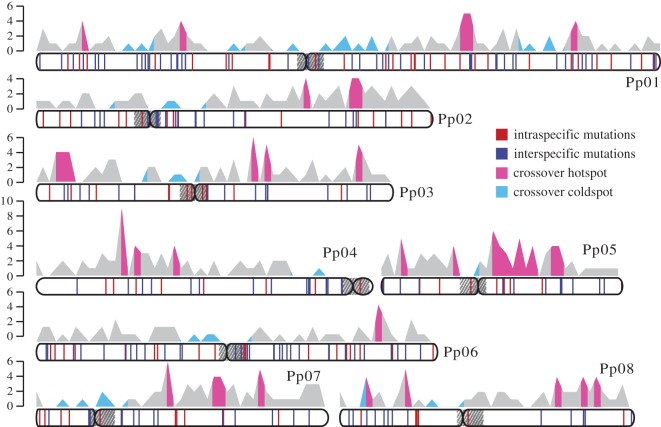
Distribution of de novo mutations and CO events on the chromosomes. The vertical red lines show the mutation distributions of intraspecific samples (*P. persica* and *P. mira*) along each chromosome, while the blue lines reflect interspecific samples (*P. persica* × *P. davidiana*). The plots above show the number of CO events within intra- (*P. persica*) and interspecific samples counted in non-overlapping 500 kbp windows. The number of COs in each window was well conserved between intra (*P. persica*) and interspecific samples (Spearman's *ρ* = 0.150, *p* = 0.00137), therefore both events were counted together to generate the overall distributions. The left vertical bars show the CO numbers. CO hotspots are marked as cyan, while coldspots are marked as sky blue.

**Figure 2.**
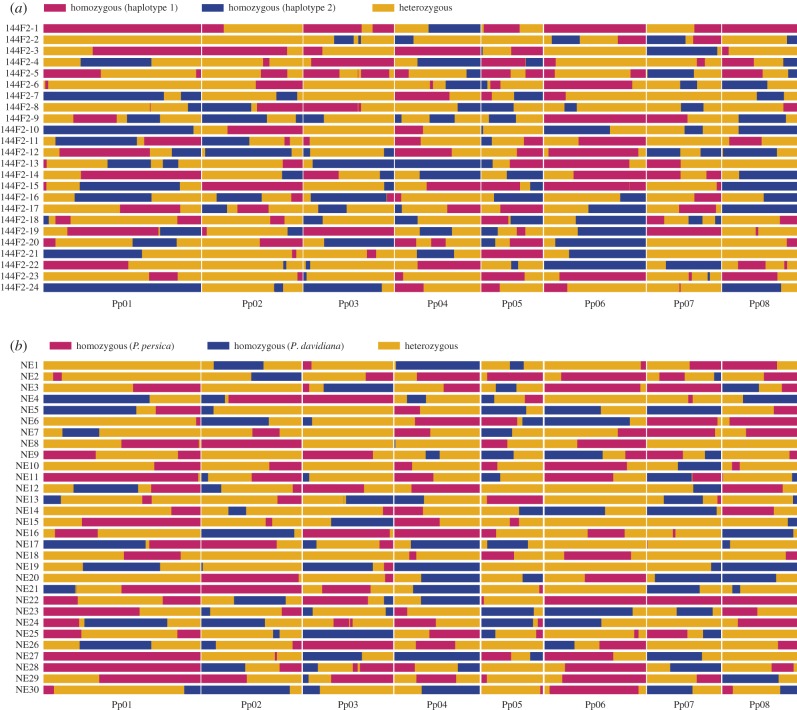
Graphical representation of CO recombinants. (*a*) Intraspecific (*P. persica*) F_2_ samples, the homozygous genotype was choosing by random for each chromosome; (*b*) interspecific F_2_ samples, the red haplotypes were derived from *P. persica* while the blue haplotypes were derived from its wild ancestor *P. davidiana*.

To identify CO events, we searched for the genotype switching point, e.g. from heterozygosity to homozygosity or from homozygosity to heterozygosity, along the chromosome pairs in each F_2_ genome [44,45] (see Material and methods for details). A total of 286 COs were detected in 24 F_2_ samples from intraspecific group I, corresponding to 11.92 COs on average or 2.64 cM Mbp^−1^ per meiosis per sample (figures 1 and 2; table 1; electronic supplementary material, tables S2–S4), which is strikingly similar to 2.61 cM Mbp^−1^ estimated from the ‘Contender’ × ‘Ambra’ F_2_ (C × A) linkage map [40].

**Table 1.** Number of COs along each chromosome.

samples	Pp01	Pp02	Pp03	Pp04	Pp05	Pp06	Pp07	Pp08	All
intraspecific groups									
mean COs	2.08	1.63	1.54	1.33	1.58	1.08	1.46	1.21	11.92
CO rate (cM Mbp^–1^)	2.18	2.67	2.82	2.58	4.28	1.76	3.26	2.68	2.64
interspecific group									
mean COs	1.60	1.27	1.23	1.10	1.17	1.00	1.07	1.03	9.47
CO rate (cM Mbp^–1^)	1.67	2.08	2.25	2.13	3.15	1.63	2.38	2.29	2.10

The CO rate of 2.6 cM Mbp^−1^ per meiosis per sample in peach is markedly lower than that in annual rice (4.53 cM Mbp^−1^) and *Arabidopsis* (4.0 cM Mbp^−1^) [45,46]. This result is consistent with previous reports of low recombination rates (0.63–2.5 cM Mbp^−1^) in other woody perennials, such as apple, pear, grape, oak and walnut, suggesting that low recombination rates may be part of the reproductive strategy of woody perennials [5].

### (c) Larger chromosomes have fewer recombination events per base pair

Among all eight chromosomes, chromosome 5 had the highest CO rate, whereas chromosome 6 had the lowest CO rate (table 1). At least in some taxa, CO rates scale inversely with chromosome size [47,48]. Consistent with this observation, a significant negative correlation was obtained between chromosome physical length and the CO rate per mega base pair (Spearman's *ρ* = −0.857, *p* = 0.0107; electronic supplementary material, figure S2). Unlike some species whose number of CO events per unit physical distance is approximately a constant [44], no positive correlation between chromosome physical length and number of CO events per chromosome was detected (Spearman's *ρ* = 0.286, *p* = 0.501).

### (d) Recombination profile is repeatable

Is the profile of recombination rate variation specific to a particular cross or repeatable between crosses? To address this we compare the variation in the recombination rate between the intra- and interspecifics groups (I and III, respectively). Despite the fact that CO number and rate varied across each chromosome, they were well correlated (Spearman's *ρ* = 0.952, *p* = 0.001) between intra- and interspecific groups (table 1; electronic supplementary material, figure S3). While the above trend reflects a between-chromosome correlation, the trend remains even if we use a small bin size of 500 kbp along each chromosome (Spearman's *ρ* = 0.150, *p* = 0.001).

The repeatability may have a simple explanation, namely that it is an artefact of stereotypical recombination rates at centromeres and telomeres. When tested using 500 kbp windows as above, the consistency persists after excluding centromeric regions (Spearman's *ρ* = 0.134, *p* = 0.00578) or both centromeric and telomeric regions (Spearman's *ρ* = 0.132, *p* = 0.00796). The telomeric regions were defined as the first and last window of each chromosome. The telomeric regions have an overall average CO rate of 1.38 cM Mbp^−1^ among groups I and III, lower than the genome average. Owing to the high correlation, we did not further distinguish intra- and inter-groups when analysing locations of hotspots and coldspots.

### (e) Peach has hotspots and coldspots of recombination

The CO events in peach were unevenly distributed on the chromosomes. The CO rate varied between 0 and 16.67 cM Mbp^−1^ when measured in non-overlapping 500 kbp windows across each chromosome (figure 1; electronic supplementary material, tables S5 and S6). We defined hotspots and coldspots by reference to randomizations (see Material and methods). We detected a total of 26 CO hotspot regions (10 000 randomizations, *p* < 0.05; electronic supplementary material, table S5) with a combined span of approximately 19-Mbp and 14 CO coldspots (10 000 randomizations, *p* < 0.05; electronic supplementary material, table S6), with a combined length of 53.8 Mbp. In other words, approximately 29% of CO events are clustered within approximately 8.6% of the entire genome (electronic supplementary material, table S5), and approximately 23.9% of the genome is devoid of the CO events (electronic supplementary material, table S6). The average recombination rate in hotspot regions (8.04 cM Mb^−1^) is about 16.8-fold higher than that in the coldspot regions (0.48 cM Mb^−1^; *t*-test, *p* = 1.58 × 10^−17^). Gene ontology analysis reveals a slight enrichment in serine-type endopeptidase activity under molecular function near hotspots (electronic supplementary material, figure S4), while coldspots are enriched for cysteine-type peptidase activity or other various binding activities, and most genes were related to the macromolecule metabolic process (electronic supplementary material, figure S5).

In contrast to prior observations in the peach genome paper [40], we observed suppression of CO in peri-centromeric regions. Among all 14 CO cold regions detected, eight were found to overlap with the putative peri-centromeric regions of all eight chromosomes (electronic supplementary material, table S6).

### (f) No evidence for higher recombination rates in domesticated peach compared with wild relatives

If domestication leads to increased recombination rates, we expect that the intraspecific cross of the domesticated peach (group I) to have a higher recombination rate than an intraspecific cross employing wild peach (group II). A conservative estimation method (see Material and methods for details), predicts an average of 3.18 cM Mbp^−1^ CO rate in wild peach (group II). Importantly, this is higher, not lower, than its domesticated relative *P. persica* (2.64 cM Mbp^−1^). The CO rate (3.02 cM Mbp^−1^) of a cross between peach and *Prunus ferganensis*, another wild undomesticated peach (virtually undistinguishable from *P. persica* at molecular level), is also higher [40].

One cross has a lower CO rate than the domesticated cross (group I), this being the interspecific cross (group III). A total of 284 COs were detected in 30 interspecific F_2_ samples (table 1), corresponding to 9.47 COs on average or 2.10 cM Mbp^−1^ per meiosis per sample, which is significantly lower than that (2.61 cM Mbp^−1^) in the intraspecific samples (Brunner Munzel test, *p* = 0.04), and also is lower than the previous estimation (3.02 cM Mbp^−1^) in the interspecific peach map of *P. persica* × *P. ferganensis* BC_1_ (P × F) [40]. The recombination reduction in interspecifics is seen in all eight chromosomes (table 1). The suppression of recombination could have resulted from decreased DNA mismatch repair activity between two diverged haplotypes [49]. Given the possibility of recombination suppression owing to the nature of the cross, we suggest that it is inappropriate to consider the group III–group I comparison when considering the domestication–recombination hypothesis.

### (g) No evidence for a correlation between recombination and mutation

While we find no increased recombination in domesticated peach, it remains interesting to ask whether recombination and mutation are coupled. Despite the abundant intragenomic variation in recombination rate, we observe no significant relationship, regardless of the bin size, between CO rate and mutation rate (500 kbp bin: Spearman's *ρ* = 0.0231, *p* = 0.636; 1 Mbp bin: *ρ* = 0.461, *p* = 0.505; 2 Mbp bin: *ρ* = 0.107, *p* = 0.275; 5 Mbp: *ρ* = 0.00317, *p* = 0.984). This mode of analysis however, may well be too crude if recombination-induced mutations are rare. Prior evidence looked for an excess of mutations within 2 kbp of recombination breakpoints [24]. In peach, however, no mutation was observed near the break points, even allowing for a more generous definition of proximity (less than 10 kbp). The nearest mutation was about 12 kbp, and only four mutations were found within 100 kbp (1 within 24 kbp and 2 within approx. 90 kbp). We conclude that we find no evidence for a coupling between mutation and recombination.

## 4. Discussion

Recent evidence, through sequencing in the vicinity of recombination break points, has found evidence that in humans [24], yeast [25] and bees [1] recombination may well be weakly mutagenic. That we failed to detect any coupling between recombination and mutation, suggests that any effect is modest at best or that peach may be unusual (perhaps domestication somehow affects this).

In many species, there is a correlation between heterozygozity and the recombination rate [50,51]. While this is classically considered a consequence of reduced Hill Robertson interference [52] in domains of high recombination, mutagenic recombination [1,24,25] is, at least in theory an alternative possibility [51,53]. In peach, we unusually do not observe a correlation between intraspecific diversity and recombination rate (500 kbp windows, *p* = 0.98, *ρ* = −0.001; 1 Mbp windows, *p* = 0.32, *ρ* = 0.084). It might then be tempting to speculate that an absence of this correlation might be coupled to the absence of mutagenic recombination and hence in those taxa with the correlation it could be owing to recombinogenic mutation. We caution against this interpretation. First, in the taxa in which recombination appears to be mutagenic the effect appears to be far too weak to explain the recombination–mutation correlation, although this will require quantitative modelling to confirm. Second, the absence of the heterozygozity–recombination correlation may have a simpler explanation, namely it is a result of domestication. Indeed, all the above results come with the caveat that peach, being a domesticated species, need not be representative and further analysis of different taxa is needed to judge the generalizability of any results.

We also fail to find evidence that domestication in this plant has led to increased recombination rates. This latter result inclines support to the view that the prior discrepancy (indirect estimation in plants supportive [30], direct evidence in mammals not supportive [32]) is owing to methodological limitations of indirect inference of recombination rather than a plant–mammal difference. One might alternatively conjecture that domestication of peach may somehow be atypical. With a sample size of one we do not wish to advocate strongly.

One notable result is the strong agreement on the local recombination rate observed between different crosses. This is not simply owing to stereotypical rates at telomeres and centromeres. This suggests that the recombinational profile of peach is relatively fixed. One might conjecture that this is as expected in a species lacking PRDM9, as hotspots defined by a mechanism dependent on PRDM9 tend to relocate over relatively short time spans, while non-PRDM9 ones do not [54–56].

## Supplementary Material

Paper II.Supp.Figures.and.Tables-20160812.docx

## Supplementary Material

Paper II.Supp.Tables-3&4.xls

## Supplementary Material

Supplementary Methods;Paper II.Supplementary Methods-20160810.docx

## Data accessibility

The sequence data from this study have been submitted to the NCBI BioProject database (http://www.ncbi.nlm.nih.gov/bioproject/) under accession no. SRP071980.

## References

[1] Yang S, Wang L, Huang J, Zhang X, Yuan Y, Chen J-Q, Hurst LD, Tian D 2015 Parent–progeny sequencing indicates higher mutation rates in heterozygotes. Nature 523, 463–467. (10.1038/nature14649) 26176923

[2] Hodgkinson A, Eyre-Walker A 2011 Variation in the mutation rate across mammalian genomes. Nat. Rev. Genet. 12, 756–766. (10.1038/nrg3098) 21969038

[3] Yue J-X, Li J, Wang D, Araki H, Tian D, Yang S 2010 Genome-wide investigation reveals high evolutionary rates in annual model plants. BMC Plant Biol. 10, 242 (10.1186/1471-2229-10-242) 21062446 PMC3095324

[4] Smith SA, Donoghue MJ 2008 Rates of molecular evolution are linked to life history in flowering plants. Science 322, 86–89. (10.1126/science.1163197) 18832643

[5] Luo M-C et al. 2015 Synteny analysis in rosids with a walnut physical map reveals slow genome evolution in long-lived woody perennials. BMC Genomics 16, 1 (10.1186/1471-2164-16-1) 26383694 PMC4574618

[6] Duncan FN 1915 An attempt to produce mutations through hybridization. Am. Nat. 49, 575–582. (10.1086/279502)

[7] Magni GE 1964 Origin and nature of spontaneous mutations in meiotic organisms. J. Cell. Comp. Physiol. 64, 165–171. (10.1002/jcp.1030640413) 14218581

[8] Magni GE, Borstel RCV 1962 Different rates of spontaneous mutation during mitosis and meiosis in yeast. Genetics 47, 1097–1108. 17248123 10.1093/genetics/47.8.1097 PMC1210391

[9] Burt A, Bell G 1987 Mammalian chiasma frequencies as a test of two theories of recombination. Nature 326, 803–805. (10.1038/326803a0) 3574451

[10] Rees H, Dale PJ 1974 Chiasmata and variability in *Lolium* and *Festuca* populations. Chromosoma 47, 335–351. (10.1007/BF00328866)

[11] Otto SP, Barton NH 2001 Selection for recombination in small populations. Evolution 55, 1921–1931. (10.1111/j.0014-3820.2001.tb01310.x) 11761054

[12] Malkova A, Haber JE 2012 Mutations arising during repair of chromosome breaks. Annu. Rev. Genet. 46, 455–473. (10.1146/annurev-genet-110711-155547) 23146099

[13] Filatov DA, Gerrard DT 2003 High mutation rates in human and ape pseudoautosomal genes. Gene 317, 67–77. (10.1016/S0378-1119(03)00697-8) 14604793

[14] Lercher MJ, Hurst LD 2002 Human SNP variability and mutation rate are higher in regions of high recombination. Trends Genet. 18, 337–340. (10.1016/S0168-9525(02)02669-0) 12127766

[15] Strathern JN, Shafer BK, McGill CB 1995 DNA synthesis errors associated with double-strand-break repair. Genetics 140, 965–972. 7672595 10.1093/genetics/140.3.965 PMC1206680

[16] Perry J, Ashworth A 1999 Evolutionary rate of a gene affected by chromosomal position. Curr. Biol. 9, 987–9S3. (10.1016/S0960-9822(99)80430-8) 10508587

[17] Nachman MW 2001 Single nucleotide polymorphisms and recombination rate in humans. Trends Genet. 17, 481–485. (10.1016/S0168-9525(01)02409-X) 11525814

[18] Aquadro CF, Bauer DuMont V, Reed FA 2001 Genome-wide variation in the human and fruitfly: a comparison. Curr. Opin. Genet. Dev. 11, 627–634. (10.1016/S0959-437X(00)00245-8) 11682305

[19] Betancourt AJ, Presgraves DC 2002 Linkage limits the power of natural selection in *Drosophila*. Proc. Natl Acad. Sci. *USA* 99, 13 616–13 620. (10.1073/pnas.212277199) 12370444 PMC129723

[20] Seplyarskiy VB et al. 2014 Crossing-over in a hypervariable species preferentially occurs in regions of high local similarity. Mol. Biol. Evol. 31, 3016–3025. (10.1093/molbev/msu242) 25135947 PMC4209137

[21] The 1000 Genomes Project Consortium 2010 A map of human genome variation from population-scale sequencing. Nature 467, 1061–1073. (10.1038/nature09534) PMC3042601 20981092

[22] Duret L, Arndt PF 2008 The impact of recombination on nucleotide substitutions in the human genome. PLoS Genet. 4, e1000071 (10.1371/journal.pgen.1000071) 18464896 PMC2346554

[23] Webster MT, Hurst LD 2012 Direct and indirect consequences of meiotic recombination: implications for genome evolution. Trends Genet. 28, 101–109. (10.1016/j.tig.2011.11.002) 22154475

[24] Arbeithuber B, Betancourt AJ, Ebner T, Tiemann-Boege I 2015 Crossovers are associated with mutation and biased gene conversion at recombination hotspots. Proc. Natl Acad. Sci. *USA* 112, 2109–2114. (10.1073/pnas.1416622112) 25646453 PMC4343121

[25] Rattray A, Santoyo G, Shafer B, Strathern JN 2015 Elevated mutation rate during meiosis in *Saccharomyces cerevisiae*. PLoS Genet. 11, e1004910 (10.1371/journal.pgen.1004910) 25569256 PMC4287439

[26] Sung W, Ackerman MS, Gout J-F, Miller SF, Williams E, Foster PL, Lynch M 2015 Asymmetric context-dependent mutation patterns revealed through mutation–accumulation experiments. Mol. Biol. Evol. 32, 1672–1683. (10.1093/molbev/msv055) 25750180 PMC4476155

[27] Tian D et al. 2008 Single-nucleotide mutation rate increases close to insertions/deletions in eukaryotes. Nature 455, 105–108. (10.1038/nature07175) 18641631

[28] Stamatoyannopoulos JA, Adzhubei I, Thurman RE, Kryukov GV, Mirkin SM, Sunyaev SR 2009 Human mutation rate associated with DNA replication timing. Nat. Genet. 41, 393–395. (10.1038/ng.363) 19287383 PMC2914101

[29] Makova KD, Hardison RC 2015 The effects of chromatin organization on variation in mutation rates in the genome. Nat. Rev. Genet. 16, 213–223. (10.1038/nrg3890) 25732611 PMC4500049

[30] Ross-Ibarra J 2004 The evolution of recombination under domestication: a test of two hypotheses. Am. Nat. 163, 105–112. (10.1086/380606) 14767840

[31] Gornall RJ 1983 Recombination systems and plant domestication. Biol. J. Linn. Soc. 20, 375–383. (10.1111/j.1095-8312.1983.tb01598.x)

[32] Muñoz-Fuentes V et al. 2015 Strong artificial selection in domestic mammals did not result in an increased recombination rate. Mol. Biol. Evol. 32, 510–523. (10.1093/molbev/msu322) 25414125 PMC4298180

[33] DePristo MA et al. 2011 A framework for variation discovery and genotyping using next-generation DNA sequencing data. Nat. Genet. 43, 491–498. (10.1038/ng.806) 21478889 PMC3083463

[34] Abyzov A, Urban AE, Snyder M, Gerstein M 2011 CNVnator: an approach to discover, genotype, and characterize typical and atypical CNVs from family and population genome sequencing. Genome Res. 21, 974–984. (10.1101/gr.114876.110) 21324876 PMC3106330

[35] Ye K, Schulz MH, Long Q, Apweiler R, Ning Z 2009 Pindel: a pattern growth approach to detect break points of large deletions and medium sized insertions from paired-end short reads. Bioinformatics 25, 2865–2871. (10.1093/bioinformatics/btp394) 19561018 PMC2781750

[36] Chen K et al. 2009 BreakDancer: an algorithm for high-resolution mapping of genomic structural variation. Nat. Methods 6, 677–681. (10.1038/nmeth.1363) 19668202 PMC3661775

[37] Cao K et al. 2014 Comparative population genomics reveals the domestication history of the peach, *Prunus persica*, and human influences on perennial fruit crops. Genome Biol. 15, 319 (10.1186/s13059-014-0415-1) PMC4174323 25079967

[38] Li H, Durbin R 2009 Fast and accurate short read alignment with Burrows-Wheeler transform. Bioinformatics 25, 1754–1760. (10.1093/bioinformatics/btp324) 19451168 PMC2705234

[39] R Development Core Team. 2013 R: a language and environment for statistical computing. Vienna, Austria: R Foundation for Statistical Computing (http://www.R-project.org)

[40] The International Peach Genome Initiative *et al.* 2013 The high-quality draft genome of peach (*Prunus persica*) identifies unique patterns of genetic diversity, domestication and genome evolution. Nat. Genet. 45, 487–494. (10.1038/ng.2586) 23525075

[41] Foulongne M, Pascal T, Arús P, Kervella J 2003 The potential of *Prunus davidiana* for introgression into peach [*Prunus persica* (L.) Batsch] assessed by comparative mapping. Theor. Appl. Genet. 107, 227–238. (10.1007/s00122-003-1238-8) 12845438

[42] Maliepaard C et al. 1998 Aligning male and female linkage maps of apple (*Malus pumila* Mill.) using multi-allelic markers. Theor. Appl. Genet. 97, 60–73. (10.1007/s001220050867)

[43] Adam-Blondon A-F, Roux C, Claux D, Butterlin G, Merdinoglu D, This P 2004 Mapping 245 SSR markers on the *Vitis vinifera* genome: a tool for grape genetics. Theor. Appl. Genet. 109, 1017–1027. (10.1007/s00122-004-1704-y) 15184982

[44] Liu H, Zhang X, Huang J, Chen J-Q, Tian D, Hurst LD, Yang S 2015 Causes and consequences of crossing-over evidenced via a high-resolution recombinational landscape of the honey bee. Genome Biol. 16, 15 (10.1186/s13059-014-0566-0) 25651211 PMC4305242

[45] Si W et al. 2015 Widely distributed hot and cold spots in meiotic recombination as shown by the sequencing of rice F2 plants. New Phytol. 206, 1491–1502. (10.1111/nph.13319) 25664766

[46] Yang S, Yuan Y, Wang L, Li J, Wang W, Liu H, Chen J-Q, Hurst LD, Tian D 2012 Great majority of recombination events in *Arabidopsis* are gene conversion events. Proc. Natl Acad. Sci. *USA* 109, 20 992–20 997. (10.1073/pnas.1211827110) PMC3529023 23213238

[47] Kaback DB, Guacci V, Barber D, Mahon JW 1992 Chromosome size-dependent control of meiotic recombination. Science 256, 228–232. (10.1126/science.1566070) 1566070

[48] Kaback DB 1996 Chromosome–size dependent control of meiotic recombination in humans. Nat. Genet. 13, 20–21. (10.1038/ng0596-20) 8673097

[49] Li L, Jean M, Belzile F 2006 The impact of sequence divergence and DNA mismatch repair on homeologous recombination in *Arabidopsis*. Plant J. 45, 908–916. (10.1111/j.1365-313X.2006.02657.x) 16507082

[50] Jaramillo-Correa JP, Verdú M, González-Martínez SC 2010 The contribution of recombination to heterozygosity differs among plant evolutionary lineages and life-forms. BMC Evol. Biol. 10, 22 (10.1186/1471-2148-10-22) 20100325 PMC2826329

[51] Cutter AD, Payseur BA 2013 Genomic signatures of selection at linked sites: unifying the disparity among species. Nat. Rev. Genet. 14, 262–274. (10.1038/nrg3425) 23478346 PMC4066956

[52] Hill WG, Robertson A 1966 The effect of linkage on limits to artificial selection. Genet. Res. 8, 269–294. (10.1017/S0016672300010156) 5980116

[53] Hellmann I, Ebersberger I, Ptak SE, Pääbo S, Przeworski M 2003 A neutral explanation for the correlation of diversity with recombination rates in humans. Am. J. Hum. Genet. 72, 1527–1535. (10.1086/375657) 12740762 PMC1180312

[54] Lichten M 2015 Putting the breaks on meiosis. Science 350, 913 (10.1126/science.aad5404) 26586748

[55] Lam I, Keeney S 2015 Nonparadoxical evolutionary stability of the recombination initiation landscape in yeast. Science 350, 932–937. (10.1126/science.aad0814) 26586758 PMC4656144

[56] Singhal S et al. 2015 Stable recombination hotspots in birds. Science 350, 928–932. (10.1126/science.aad0843) 26586757 PMC4864528

