# Supplementary material for: Mutation rate analysis via parent–progeny sequencing of the perennial peach. II. No evidence for recombination-associated mutation
Source: Proc Biol Sci. 2016 Oct 26;283(1841):20161785. doi: 10.1098/rspb.2016.1785 (PMC5095386; doi:10.1098/rspb.2016.1785)
Supplement: Paper II.Supp.Figures.and.Tables-20160812.docx [file rspb20161785supp1.docx]

**Supplementary Figs 1-7 and supplementary tables 1,2,5,6 for**

**Mutation rate analysis via parent-progeny sequencing of the perennial peach II: No evidence for recombination-associated mutation**

Long Wang^1,3^, Yanchun Zhang^1,3^, Chao Qin^1^, Dacheng Tian^1^, Sihai Yang^1,4^, Laurence D. Hurst^2,4^

Proceedings of the Royal Society, London, Series B

Doi: 10.1098/rspb.2016.1785

Supplemental Fig S1**. Schematic diagram of genotype assignment in intraspecific (*P. persica*) F_2_ samples. A)** Blocks formed only according to homozygous and heterozygous genotypes. The A/T characters stand for different nucleotide alleles in each marker sites. Switch points (i.e. the putative break points) between different genotypes were marked by dashed lines. In the initial stage, only the homozygous and heterozygous genotypes were distinguished, and the heterozygous markers were not phased to each haplotype. **B)** Phasing all other samples by comparing to Sample1 which was homozygous across the whole chromosome. The haplotype of Sample1 was defined as the “Haplotype1”, while another haplotype in the F_1_ chromosome was defined as “Haplotype2”. The Sample5 contain two crossovers very near to each other, which would be unobservable in the initial clustering stage, but could be recovered after each haplotype was phased (shown by double dashed lines). No such situation was observed in current data, indicating a very low chance of happening. **C)** Phasing all other samples by comparing to Sample5. This stands for the situation which the pre-assumption was not hold, i.e. two crossovers happed very near to each other and was unobserved in the selected sample. After phasing, multiple independent crossovers in different samples (e.g. double dashed lines in Sample1 and Sample4 in the figure) would be observed, which was less likely to be true. This error could then be corrected by either introduce crossovers in Sample5 or choose another sample (e.g. Sample1) as the “Haplotype1”. In practice, almost all chromosomes could be easily phased. Those complex situations were merely observed.


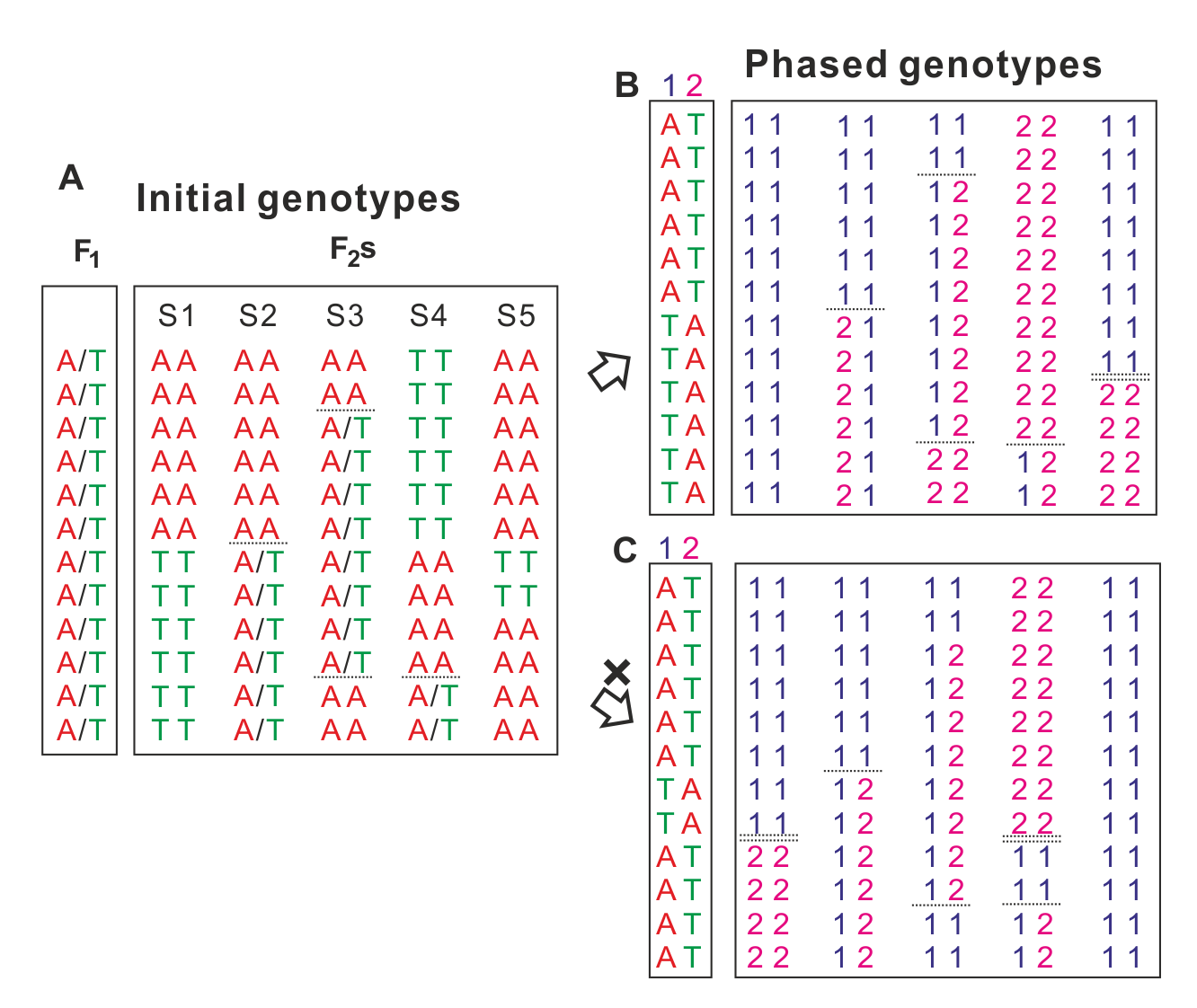


**Supplemental** **Fig S2. The relationship of overall crossover rate and chromosome size.** The dashed line represents standard linear regression and is for illustrative purposes only.


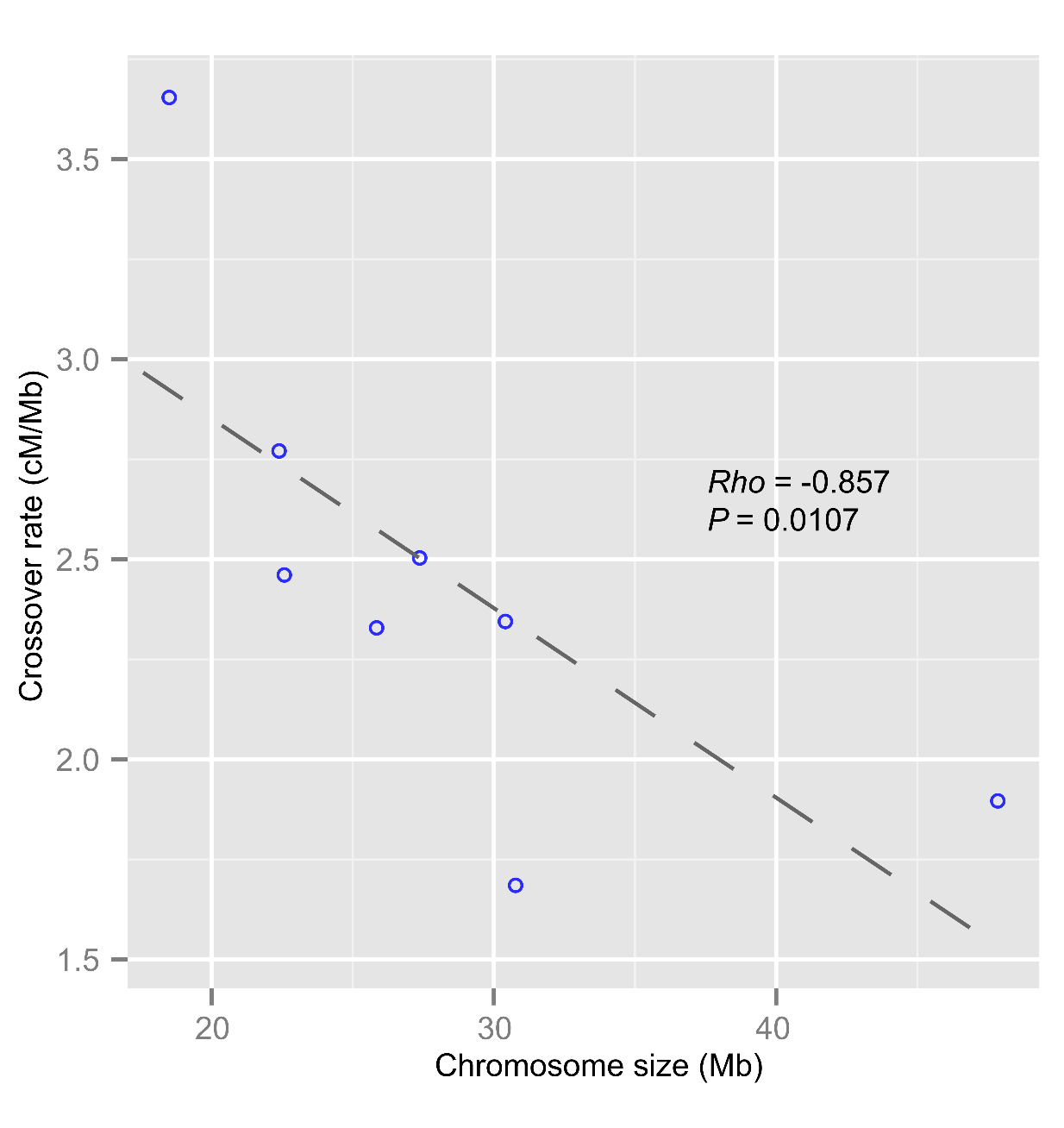


**Supplemental** **Fig S3. The correlation of (A) average crossover numbers or (B) average crossover rate between intra- (*P. persica*) and inter- specific groups.** The dashed line represents standard linear regression and is for illustrative purposes only.


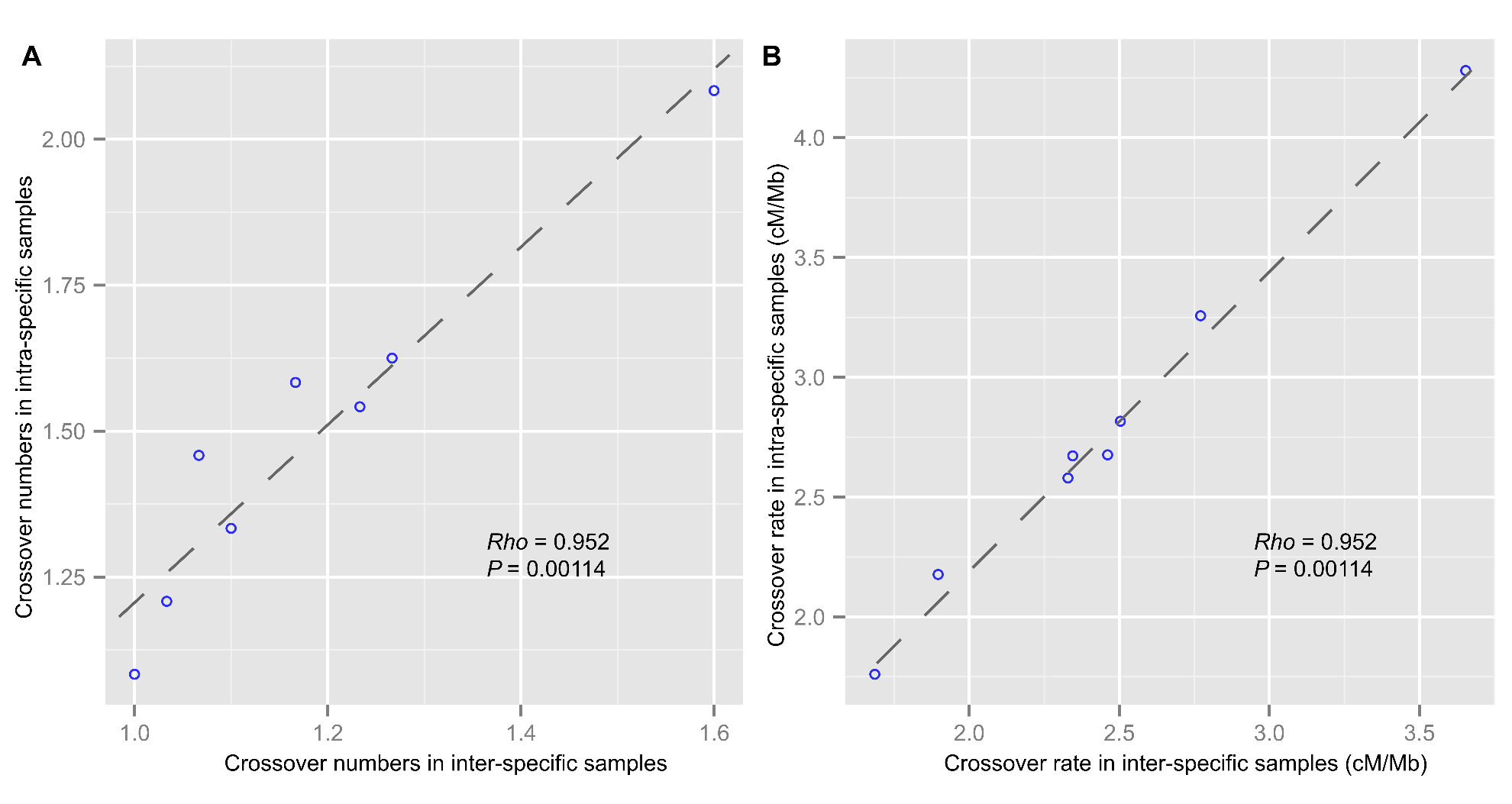


**Supplemental** **Fig S4. Enrichment of gene ontology (GO) terms of the genes within the recombination hotspot regions.** Enrichment test and graphic presentation were performed using the Biological Networks Gene Ontology tool (BiNGO) [1]. Gene ontology file were downloaded from Gene Ontology Consortium database (<http://purl.obolibrary.org/obo/go/go-basic.obo>, data-version: 2015-11-14), functional annotation for peach genome were downloaded from GDR database (<https://www.rosaceae.org/species/prunus_persica/>, version 2.0.a1). The significance *P*-values were obtained through hypergeometric test after corrected for multiple testing using Benjamini & Hochberg False Discovery Rate (FDR) correction implemented in BiNGO.


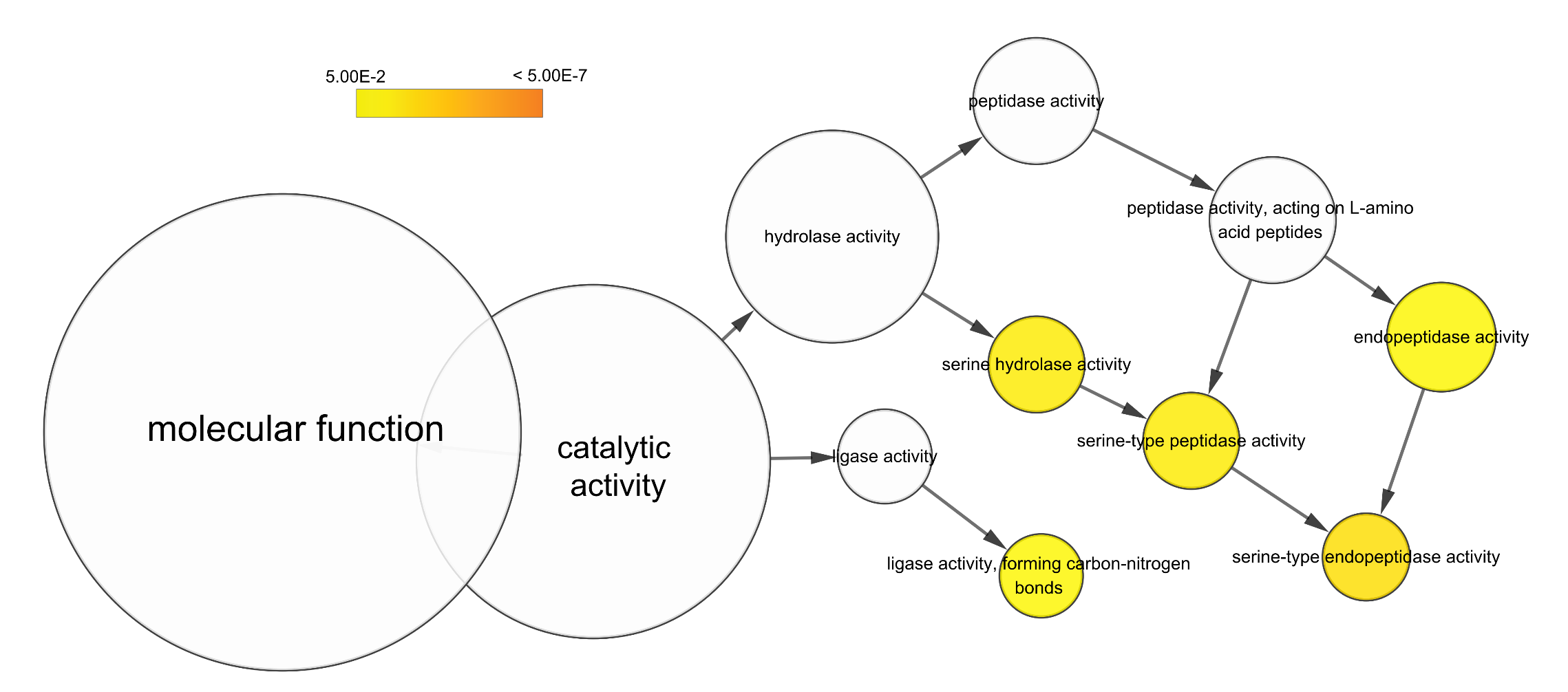


**Supplemental** **Fig S5. Enrichment of GO terms of the genes within the recombination coldspot regions.**

**
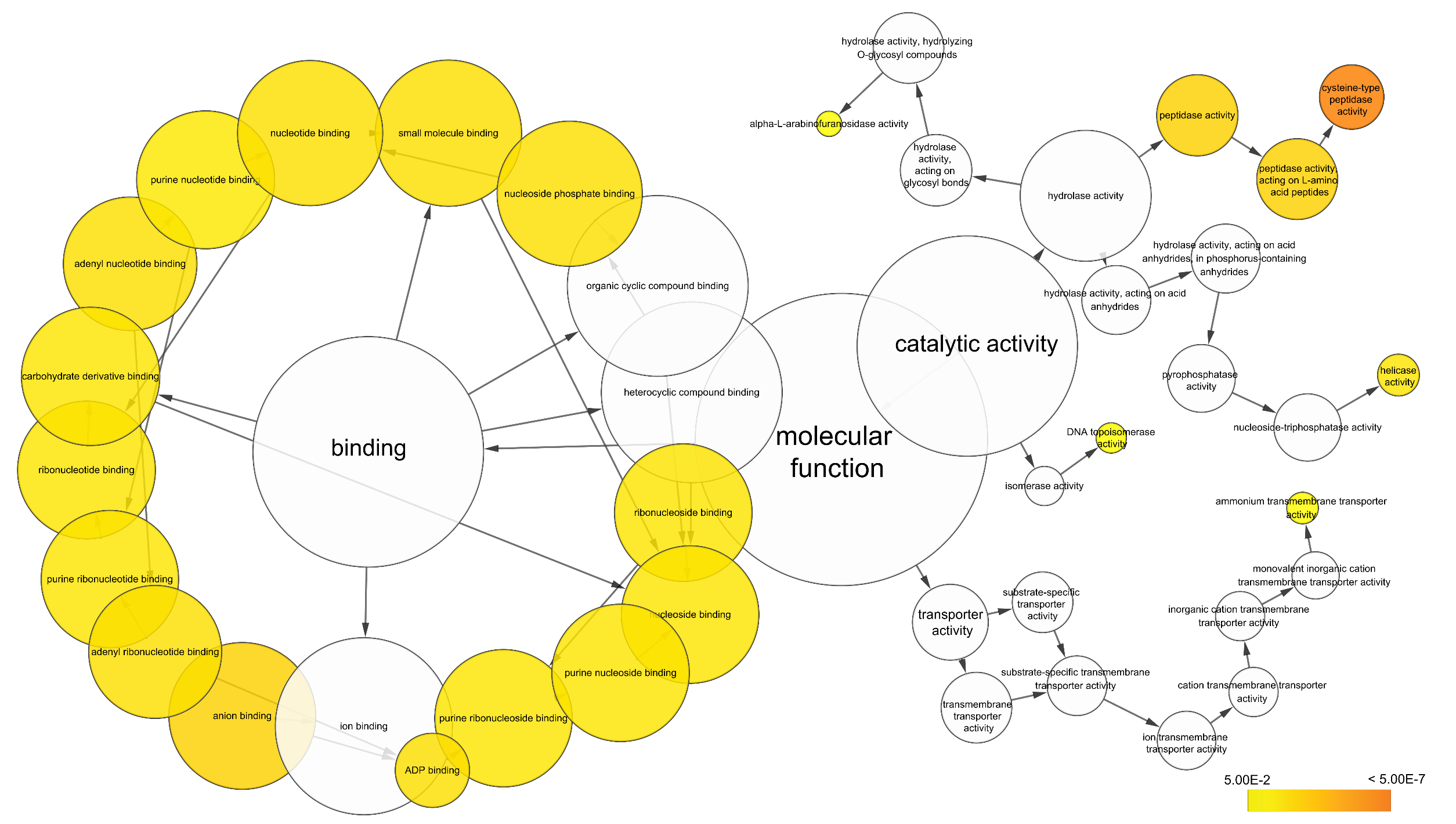
(A)** GO for molecular function

**
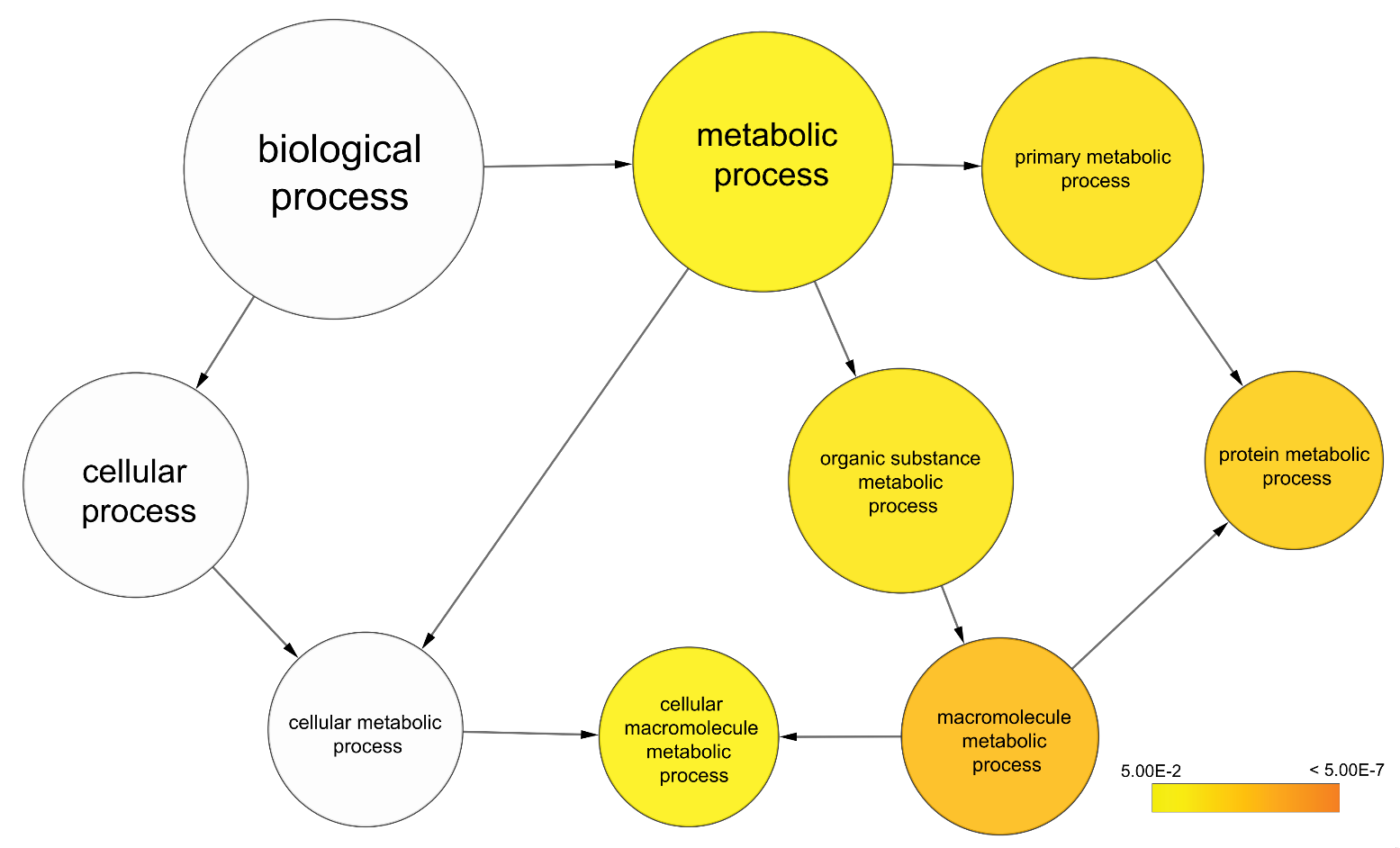
(B)** GO for biological process

Supplemental Fig S6**.** **Sampling timeline of intraspecific group I and interspecific group III.** All trees were sampled in 2015. Leaves were collected from an arbitrary branch of a tree or from the whole saplings.

**
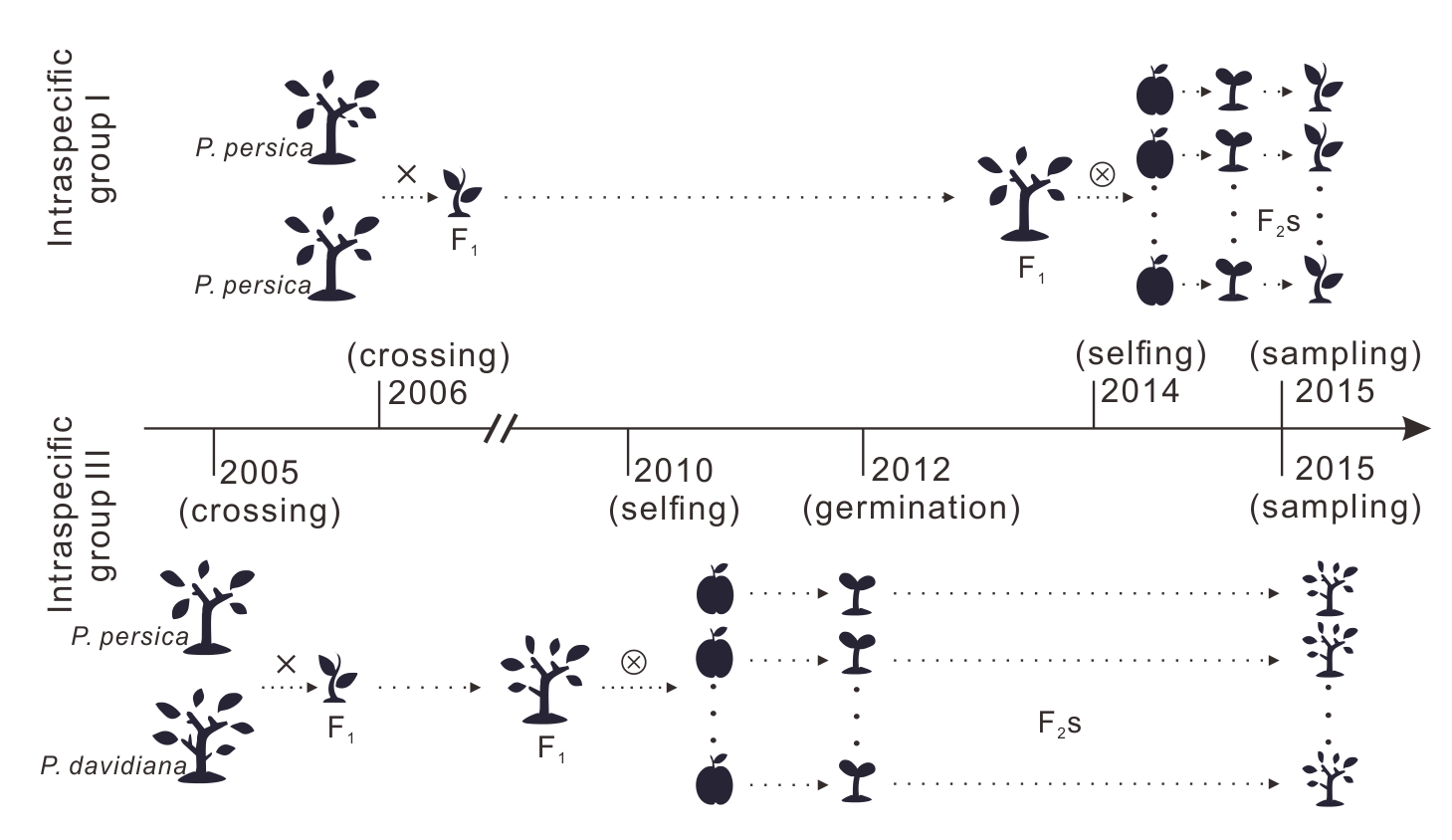
**

Supplemental Fig S7**.** **Pedigree information of peach F_2_s of the interspecific group (III).** In this pedigree, the 2005-W is the F_1_ offspring of ZXST-1 (*Prunus* *davidiana*) and 96-7-52 (*Prunus persica*). In this pedigree, 30 selfed F_2_s (NE1 to 30) from a F_1_ tree of 2005-W were employed to detect *de novo* mutations and crossover events. In addition, except for 91-1-5 and 96-7-52 which have already died, the other four parents (HR-E, MLWL-E, SG-E and ZXST-1 have been sequenced in this study. HR-E (Sunred), *Prunus persica* (L.) Batsch; MLWL-E (Maravilha), *Prunus persica* (L.) Batsch. SG-E (Shu Guang) *Prunus persica* (L.) Batsch; ZXST-1, (Zhou Xing Shan Tao), *Prunus* *davidiana*(Carr.)　Franch.


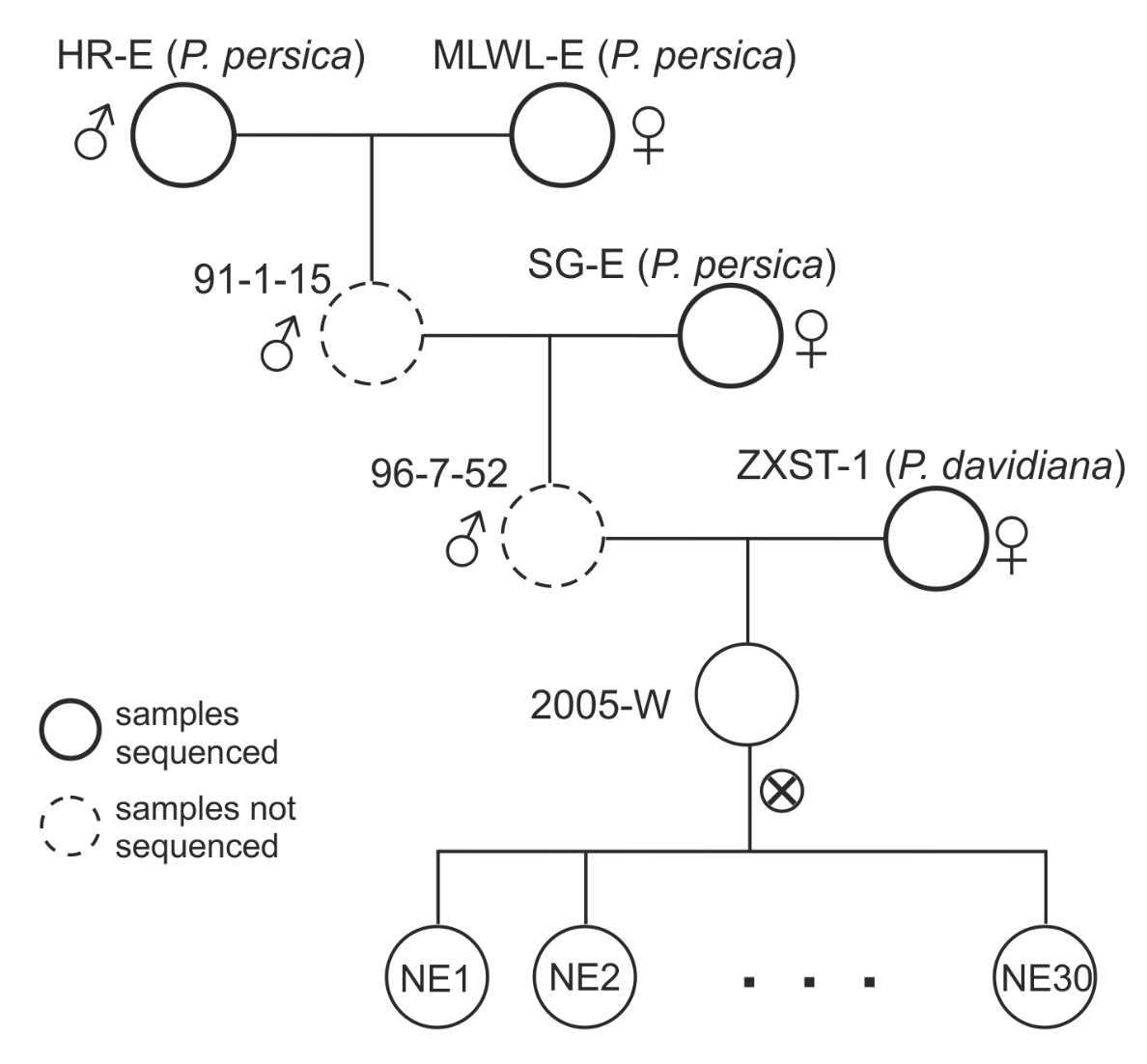


**Supplemental** **Table S1. Sequencing depth and genome coverage (%) of all samples.**

| **Type** | **Sample** | **Clean data (Gb)** | **Sequencing depth** | **Uniquely mapped depth*** | **Uniquely mapped coverage (≥5 reads)** |
| --- | --- | --- | --- | --- | --- |
| **Intraspecific groups** | | | | | |
| *P. persica* F_1_ sample | 144F1-3 | 11.0 | 48.4 | 31.7 | 93.3 |
| *P. persica* F_2_ samples | 144F2-1 | 10.6 | 46.6 | 29.9 | 90.8 |
|  | 144F2-2 | 12.5 | 54.9 | 35.0 | 92.8 |
|  | 144F2-3 | 11.1 | 48.7 | 31.0 | 91.1 |
|  | 144F2-4 | 11.6 | 51.2 | 32.9 | 92.2 |
|  | 144F2-5 | 11.7 | 51.4 | 33.9 | 91.9 |
|  | 144F2-6 | 11.7 | 51.6 | 33.0 | 92.3 |
|  | 144F2-7 | 11.8 | 51.8 | 33.7 | 92.8 |
|  | 144F2-8 | 10.1 | 44.5 | 28.7 | 91.3 |
|  | 144F2-9 | 10.3 | 45.4 | 28.1 | 91 |
|  | 144F2-10 | 12.3 | 54.0 | 33.8 | 92.9 |
|  | 144F2-11 | 12.2 | 53.8 | 33.2 | 92 |
|  | 144F2-12 | 14.5 | 63.6 | 38.1 | 90.9 |
|  | 144F2-13 | 10.8 | 47.3 | 30.8 | 92.5 |
|  | 144F2-14 | 10.4 | 45.9 | 28.2 | 91.4 |
|  | 144F2-15 | 11.8 | 51.9 | 32.1 | 91.8 |
|  | 144F2-16 | 10.2 | 45.0 | 27.0 | 91.8 |
|  | 144F2-17 | 10.7 | 46.8 | 29.7 | 92.2 |
|  | 144F2-18 | 12.3 | 54.0 | 32.2 | 92.5 |
|  | 144F2-19 | 12.8 | 56.4 | 32.2 | 91.7 |
|  | 144F2-20 | 12.1 | 53.1 | 31.9 | 92.6 |
|  | 144F2-21 | 11.5 | 50.6 | 30.7 | 92.8 |
|  | 144F2-22 | 11.0 | 48.2 | 28.4 | 91.7 |
|  | 144F2-23 | 11.2 | 49.4 | 30.5 | 92.3 |
|  | 144F2-24 | 11.0 | 48.4 | 30.0 | 92.8 |
| *P. mira* F_1_ sample | GZ-1 | 10.67 | 46.9 | 26.6 | 78 |
| *P. mira*  F_2_ samples | GZTH-5 | 11.32 | 49.8 | 25.3 | 77.4 |
|  | GZTH-8** | 11.94 | 52.5 | 14.3 | 74.9 |
|  | GZTH-S1 | 10.04 | 44.1 | 26.8 | 76.6 |
|  | GZTH-S2 | 8.72 | 38.3 | 23.3 | 76.7 |
|  | GZTH-S3 | 11.78 | 51.8 | 30.1 | 77.4 |
|  | GZTH-S4 | 12.07 | 53.1 | 31.6 | 77.3 |
|  | GZTH-S5 | 12.16 | 53.5 | 21.7 | 76.8 |
|  | GZTH-S7 | 10.48 | 46.1 | 27.5 | 77.2 |
|  | GZTH-S8 | 11.94 | 52.5 | 31.6 | 77.9 |
|  | GZTH-S9 | 10.83 | 47.6 | 28.6 | 77.6 |
| **Interspecific group (*P. persica* × *P. davidiana*)** | | | | | |
| Parent Samples | HR-E | 10.9 | 47.8 | 35.3 | 92.7 |
|  | MLWL-E | 10.5 | 46.2 | 32.9 | 93.2 |
|  | SG-E | 12.2 | 53.8 | 38.6 | 94.7 |
|  | ZXST-1 | 12.5 | 54.8 | 32.8 | 79.5 |
| Heterozygous F_1_ sample | 2005-W | 11.6 | 50.9 | 33.3 | 92.4 |
| Heterozygous F_2_ samples | NE1 | 13.5 | 59.2 | 37.0 | 89.1 |
|  | NE2 | 11.1 | 48.9 | 32.8 | 91.8 |
|  | NE3 | 12.3 | 53.9 | 34.9 | 88.8 |
|  | NE4 | 12.5 | 55.1 | 32.4 | 87 |
|  | NE5 | 11.5 | 50.4 | 32.2 | 86 |
|  | NE6 | 12.0 | 52.9 | 32.1 | 87.6 |
|  | NE7 | 11.7 | 51.5 | 33.5 | 90.2 |
|  | NE8 | 11.4 | 50.3 | 31.4 | 91.3 |
|  | NE9 | 11.5 | 50.6 | 33.3 | 89.5 |
|  | NE10 | 12.1 | 53.1 | 34.6 | 91.8 |
|  | NE11 | 12.2 | 53.8 | 34.8 | 90.7 |
|  | NE12 | 12.0 | 52.6 | 35.1 | 89.6 |
|  | NE13 | 11.2 | 49.2 | 32.2 | 90.2 |
|  | NE14 | 9.9 | 43.6 | 28.9 | 87.6 |
|  | NE15 | 12.7 | 56.0 | 34.6 | 90.3 |
|  | NE16 | 11.9 | 52.4 | 33.7 | 87.6 |
|  | NE17 | 11.9 | 52.4 | 32.4 | 87 |
|  | NE18 | 13.6 | 59.8 | 38.8 | 92.6 |
|  | NE19 | 12.5 | 55.1 | 32.8 | 85 |
|  | NE20 | 12.1 | 53.3 | 33.9 | 88.5 |
|  | NE21 | 11.8 | 52.0 | 32.0 | 89 |
|  | NE22 | 15.0 | 65.8 | 38.7 | 89.8 |
|  | NE23 | 14.3 | 62.7 | 37.9 | 88.7 |
|  | NE24 | 13.7 | 60.3 | 36.6 | 86.4 |
|  | NE25 | 13.7 | 60.3 | 37.5 | 89.9 |
|  | NE26 | 11.2 | 49.5 | 30.7 | 88.7 |
|  | NE27 | 10.4 | 45.6 | 30.2 | 87.7 |
|  | NE28 | 12.5 | 55.1 | 35.6 | 90.2 |
|  | NE29 | 11.2 | 49.3 | 33.3 | 90.4 |
|  | NE30 | 11.1 | 48.7 | 30.9 | 87.6 |

*Reads with a mapping quality ≥20 were considered as uniquely mapped;

**Sample poorly sequenced and only used in comparing to exclude false positives.

**Supplemental** **Table S2. Number of crossovers (COs) along each chromosomes**

| **Samples** | **Pp01** | **Pp02** | **Pp03** | **Pp04** | **Pp05** | **Pp06** | **Pp07** | **Pp08** | **All** |
| --- | --- | --- | --- | --- | --- | --- | --- | --- | --- |
| **Intraspecific group (*P. persica*)** | | | | | | | | | |
| 144F2-1 | 0 | 1 | 2 | 1 | 2 | 0 | 1 | 0 | 7 |
| 144F2-2 | 0 | 0 | 4 | 1 | 0 | 3 | 2 | 1 | 11 |
| 144F2-3 | 1 | 1 | 1 | 0 | 2 | 0 | 1 | 1 | 7 |
| 144F2-4 | 2 | 2 | 1 | 1 | 2 | 1 | 2 | 2 | 13 |
| 144F2-5 | 2 | 2 | 5 | 2 | 2 | 2 | 2 | 2 | 19 |
| 144F2-6 | 2 | 1 | 0 | 3 | 3 | 1 | 2 | 1 | 13 |
| 144F2-7 | 2 | 2 | 0 | 1 | 2 | 1 | 0 | 2 | 10 |
| 144F2-8 | 3 | 2 | 3 | 1 | 1 | 2 | 2 | 1 | 15 |
| 144F2-9 | 4 | 2 | 1 | 3 | 2 | 0 | 1 | 2 | 15 |
| 144F2-10 | 1 | 1 | 0 | 1 | 1 | 1 | 2 | 1 | 8 |
| 144F2-11 | 3 | 3 | 1 | 1 | 2 | 1 | 0 | 2 | 13 |
| 144F2-12 | 3 | 2 | 2 | 1 | 1 | 2 | 2 | 2 | 15 |
| 144F2-13 | 5 | 1 | 1 | 0 | 2 | 1 | 1 | 0 | 11 |
| 144F2-14 | 1 | 1 | 2 | 1 | 1 | 1 | 2 | 1 | 10 |
| 144F2-15 | 3 | 0 | 1 | 1 | 2 | 2 | 1 | 0 | 10 |
| 144F2-16 | 2 | 3 | 3 | 1 | 1 | 1 | 1 | 1 | 13 |
| 144F2-17 | 2 | 3 | 2 | 2 | 1 | 1 | 2 | 0 | 13 |
| 144F2-18 | 4 | 2 | 1 | 1 | 2 | 1 | 4 | 1 | 16 |
| 144F2-19 | 3 | 2 | 0 | 2 | 3 | 1 | 0 | 2 | 13 |
| 144F2-20 | 3 | 1 | 1 | 3 | 2 | 1 | 0 | 1 | 12 |
| 144F2-21 | 1 | 2 | 2 | 2 | 0 | 1 | 0 | 0 | 8 |
| 144F2-22 | 1 | 2 | 2 | 1 | 2 | 0 | 1 | 4 | 13 |
| 144F2-23 | 2 | 1 | 1 | 1 | 1 | 1 | 4 | 1 | 12 |
| 144F2-24 | 0 | 2 | 1 | 1 | 1 | 1 | 2 | 1 | 9 |
| Total | 50 | 39 | 37 | 32 | 38 | 26 | 35 | 29 | 286 |
| Mean COs | 2.08 | 1.63 | 1.54 | 1.33 | 1.58 | 1.08 | 1.46 | 1.21 | 11.92 |
| CO Rate (cM/Mb) | 2.18 | 2.67 | 2.82 | 2.58 | 4.28 | 1.76 | 3.26 | 2.68 | 2.64 |
| **Interspecific group** | | | | | | | | | |
| NE1 | 0 | 2 | 1 | 1 | 2 | 1 | 1 | 1 | 9 |
| NE2 | 2 | 1 | 1 | 1 | 1 | 1 | 3 | 1 | 11 |
| NE3 | 1 | 0 | 2 | 1 | 2 | 1 | 0 | 2 | 9 |
| NE4 | 1 | 2 | 0 | 2 | 2 | 0 | 2 | 1 | 10 |
| NE5 | 2 | 1 | 0 | 1 | 1 | 1 | 0 | 1 | 7 |
| NE6 | 1 | 1 | 1 | 1 | 2 | 1 | 1 | 1 | 9 |
| NE7 | 2 | 2 | 0 | 1 | 1 | 1 | 1 | 1 | 9 |
| NE8 | 2 | 0 | 0 | 1 | 1 | 1 | 0 | 0 | 5 |
| NE9 | 2 | 0 | 1 | 2 | 1 | 2 | 2 | 1 | 11 |
| NE10 | 1 | 1 | 0 | 1 | 1 | 1 | 1 | 2 | 8 |
| NE11 | 1 | 2 | 1 | 1 | 1 | 1 | 2 | 2 | 11 |
| NE12 | 3 | 2 | 1 | 1 | 0 | 0 | 1 | 1 | 9 |
| NE13 | 3 | 1 | 3 | 1 | 1 | 0 | 3 | 1 | 13 |
| NE14 | 1 | 2 | 1 | 1 | 1 | 1 | 1 | 1 | 9 |
| NE15 | 1 | 2 | 1 | 1 | 2 | 0 | 0 | 1 | 8 |
| NE16 | 2 | 1 | 1 | 0 | 1 | 2 | 2 | 1 | 10 |
| NE17 | 2 | 1 | 2 | 1 | 2 | 0 | 1 | 1 | 10 |
| NE18 | 2 | 0 | 0 | 2 | 1 | 2 | 0 | 1 | 8 |
| NE19 | 2 | 1 | 2 | 0 | 0 | 0 | 1 | 0 | 6 |
| NE20 | 0 | 1 | 1 | 1 | 1 | 1 | 1 | 1 | 7 |
| NE21 | 4 | 0 | 2 | 1 | 1 | 0 | 1 | 2 | 11 |
| NE22 | 2 | 2 | 2 | 1 | 1 | 0 | 0 | 0 | 8 |
| NE23 | 1 | 2 | 2 | 1 | 1 | 1 | 2 | 1 | 11 |
| NE24 | 3 | 1 | 4 | 1 | 2 | 1 | 0 | 1 | 13 |
| NE25 | 2 | 2 | 0 | 1 | 2 | 2 | 2 | 0 | 11 |
| NE26 | 2 | 2 | 0 | 2 | 0 | 2 | 0 | 1 | 9 |
| NE27 | 0 | 2 | 1 | 0 | 2 | 1 | 1 | 0 | 7 |
| NE28 | 0 | 2 | 4 | 2 | 0 | 1 | 1 | 2 | 12 |
| NE29 | 1 | 1 | 2 | 2 | 1 | 1 | 1 | 1 | 10 |
| NE30 | 2 | 1 | 1 | 1 | 1 | 4 | 1 | 2 | 13 |
| Total | 48 | 38 | 37 | 33 | 35 | 30 | 32 | 31 | 284 |
| Mean COs | 1.60 | 1.27 | 1.23 | 1.10 | 1.17 | 1.00 | 1.07 | 1.03 | 9.47 |
| CO Rate (cM/Mb) | 1.67 | 2.08 | 2.25 | 2.13 | 3.15 | 1.63 | 2.38 | 2.29 | 2.10 |

**Supplemental** **Table S5. Hot spot regions of the crossover events (COs).**

|  | Chrom osome | Points of genotype switching | | Length (Mb) | CO count | Recombination  rate (cM Mb^-1^) | *P*-value |
| --- | --- | --- | --- | --- | --- | --- | --- |
|  |  | Start (Mb) | End (Mb) |  |  |  |  |
| 1 | chr01 | 3.5 | 4.0 | 0.5 | 4 | 7.41 | 0.0393 |
| 2 | chr01 | 11.0 | 11.5 | 0.5 | 4 | 7.41 | 0.0376 |
| 3 | chr01 | 32.5 | 33.5 | 1.0 | 10 | 9.26 | 0.0002 |
| 4 | chr01 | 41 | 41.5 | 0.5 | 4 | 7.41 | 0.0387 |
| 5 | chr02 | 20.5 | 21.0 | 0.5 | 4 | 7.41 | 0.0388 |
| 6 | chr02 | 24.0 | 25.0 | 1.0 | 8 | 7.41 | 0.0043 |
| 7 | chr03 | 1.5 | 3.0 | 1.5 | 12 | 7.41 | 0.0011 |
| 8 | chr03 | 16.5 | 17.0 | 0.5 | 6 | 11.1 | 0.0012 |
| 9 | chr03 | 17.5 | 18 | 0.5 | 5 | 9.3 | 0.0099 |
| 10 | chr03 | 24.5 | 25.0 | 0.5 | 5 | 9.26 | 0.0089 |
| 11 | chr04 | 6.5 | 7.0 | 0.5 | 9 | 16.67 | <0.0001 |
| 12 | chr04 | 7.5 | 8.0 | 0.5 | 4 | 7.41 | 0.0353 |
| 13 | chr04 | 10.5 | 11 | 0.5 | 4 | 7.41 | 0.0395 |
| 14 | chr05 | 1.5 | 2.0 | 0.5 | 5 | 9.26 | 0.0097 |
| 15 | chr05 | 5.5 | 6.0 | 0.5 | 4 | 7.41 | 0.0374 |
| 16 | chr05 | 8.5 | 12 | 3.5 | 24 | 6.35 | 0.0001 |
| 17 | chr05 | 13.0 | 14 | 1 | 8 | 7.41 | 0.0047 |
| 18 | chr06 | 26 | 26.5 | 0.5 | 5 | 9.26 | 0.0098 |
| 19 | chr07 | 10 | 10.5 | 0.5 | 6 | 11.11 | 0.0016 |
| 20 | chr07 | 13.5 | 14.5 | 1 | 8 | 7.41 | 0.005 |
| 21 | chr07 | 17.0 | 17.5 | 0.5 | 5 | 9.26 | 0.0097 |
| 22 | chr08 | 2.0 | 2.5 | 0.5 | 4 | 7.41 | 0.038 |
| 23 | chr08 | 5.0 | 5.5 | 0.5 | 5 | 9.26 | 0.0086 |
| 24 | chr08 | 16.5 | 17.0 | 0.5 | 4 | 7.41 | 0.0341 |
| 25 | chr08 | 18.5 | 19.0 | 0.5 | 4 | 7.41 | 0.0411 |
| 26 | chr08 | 19.5 | 20.0 | 0.5 | 4 | 7.41 | 0.0392 |
| Mean | - | - | - | 0.73 | 6.35 | 8.04 | - |
| Total length | 19Mb (225Mb for all genome) | | | | | |  |

**Supplemental** **Table S6. Cold spot regions of the crossover events (COs).**

|  | Chrom osome | Points of genotype switching | | Length(Mb) | CO count | Recombination  rate (cM Mb^-1^) | *P*-value |
| --- | --- | --- | --- | --- | --- | --- | --- |
|  |  | Start (Mb) | End (Mb) |  |  |  |  |
| 1 | chr01 | 6.0 | 9.0 | 3.0 | 2 | 0.62 | 0.0186 |
| 2 | chr01 | 13.0 | 16.0 | 3.0 | 1 | 0.31 | 0.0038 |
| 3 | chr01 | 18.0 | 27.0 | 9.0* | 8 | 0.82 | <0.0001 |
| 4 | chr01 | 37.0 | 40.5 | 3.5 | 3 | 0.79 | 0.0206 |
| 5 | chr02 | 4.5 | 6.0 | 1.5 | 0 | 0 | 0.0219 |
| 6 | chr02 | 8.5 | 13.0 | 4.5* | 2 | 0.41 | 0.0014 |
| 7 | chr03 | 6.5 | 8.5 | 2.0 | 0 | 0 | 0.0065 |
| 8 | chr03 | 9.5 | 12.5 | 3* | 2 | 0.62 | 0.0181 |
| 9 | chr04 | 19.5 | 25.8 | 6.3* | 1 | 0.15 | <0.0001 |
| 10 | chr05 | 6.0 | 7.5 | 1.5* | 0 | 0 | 0.0214 |
| 11 | chr06 | 11 | 16.5 | 5.5* | 3 | 0.51 | 0.0001 |
| 12 | chr07 | 0.5 | 7.0 | 6.5* | 5 | 0.71 | 0.0009 |
| 13 | chr08 | 0.5 | 2.0 | 1.5 | 0 | 0 | 0.0229 |
| 14 | chr08 | 6.5 | 9.5 | 3.0* | 1 | 0.31 | 0.0045 |
| Mean | - | - | - | 3.84 | 2 | 0.48 | - |
| Total length | 53.8Mb (225Mb for all genome) | | | | | |  |

* indicates that this region include centromere.

**Supplementary References**

1. Maere, S., Heymans, K. & Kuiper, M. 2005 BiNGO: a Cytoscape plugin to assess overrepresentation of Gene Ontology categories in Biological Networks. *Bioinformatics* **21**, 3448–3449. (doi:10.1093/bioinformatics/bti551)
