# Supplementary material for: Mutation rate analysis via parent–progeny sequencing of the perennial peach. II. No evidence for recombination-associated mutation
Source: Proc Biol Sci. 2016 Oct 26;283(1841):20161785. doi: 10.1098/rspb.2016.1785 (PMC5095386; doi:10.1098/rspb.2016.1785)
Supplement: Supplementary Methods;Paper II.Supplementary Methods-20160810.docx [file rspb20161785supp3.docx]

**Supplementary Methods for**

**Mutation rate analysis via parent-progeny sequencing of the perennial peach II: No evidence for recombination-associated mutation**

Long Wang^1,3^, Yanchun Zhang^1,3^, Chao Qin^1^, Dacheng Tian^1^, Sihai Yang^1,4^, Laurence D. Hurst^2,4^

Proceedings of the Royal Society, London, Series B

Doi: 10.1098/rspb.2016.1785

And for

**Mutation rate analysis via parent-progeny sequencing of the perennial peach I. A low rate in woody perennials and a higher mutagenicity in hybrids**

Zhengqing Xie, Long Wang, Lirong Wang, Zhiqiang Wang, Zhenhua Lu, Dacheng Tian, Sihai Yang, Laurence D. Hurst

Proceedings of the Royal Society, London, Series B

DOI: 10.1098/rspb.2016.1016

Supplementary Methods. Full methods for papers I and II

# Methods

## a. Sampling

In order to test the relationship between mutation and hybridization as well as recombination, we constructed three parent-progeny groups (group I~III). Each group has an F_1_ parent tree together with its selfed F_2_ progeny. The F_1_ parent trees were derived from crosses either between different peach cultivars, or between different Prunus species, representing different heterozygosity. Groups I and II are intraspecific crosses employing young (group I) and old (group II) parents, while group III is an interspecific cross.

For intraspecific group (group I), one F_1_ individual was obtained from a cross between two peach (*Prunus persica*) varieties in 2006, and was selfed in 2014 to generate the F_2_ fruits (Supplemental Fig S6). The F_2_ fruits were stored at low temperature for about three months to increase germination rate. Successful germinated seeds were then grown into small trees, and leaves from 24 randomly selected F_2_ saplings (144F2-1 to -24) were sampled three months later in 2015 (Supplemental Fig S6). The parent tree was about nine years old when sampled in 2015 with one arbitrary branch being chosen to collect fresh leaves for DNA extraction.

The above intraspecific group has a young F_1_ parent and a young F_2_ progeny. To attempt to exclude a possible influence of somatic mutations on our estimation, we additionally examined an intraspecific cross with a very old parent and a young F_2_ progeny. This intraspecific cross (group II) was a wild peach (*Prunus mira* Koehne) sampled in Nyingchi, Tibet, China (latitude 29°42.018' N, longitude 94°20.560' E). The F_1_ parent tree has been growing for approximately 200~300 years. The tree was over eight meters tall and with a trunk over one meter in diameter. The tree was geographically isolated from other wild peach trees, and the fruits from this tree were assumed to be from selfing. It is unknown whether this parent tree itself was from selfing or outcrossing of different *P. mira* trees. This tree was sampled in May 2015, prior to that year’s seed set. The F_2_ seeds were collected under this tree in 2014. The sequencing data confirmed that the progeny were from selfing, as no other genetic source has been observed excepting the parent tree. Some of the F_2_ seeds were then treated with Gibberellin (GA) to enable fast germination (requiring only 1~2 weeks). Eight successfully germinated F_2_ individuals (GZTH-S1 to –S5, -S7 to –S9) were grown in a light incubator for about 2~3 months for leaf sampling (Supplemental Table S1). Two samples (GZTH-5 and GZTH-8) were not germinated and the seed was directly used as raw material after carefully removing the seed coat. One sample GZTH-8 was highly contaminated and sequenced with low quality. The sequencing data from this sample was thus only used to enable removal of false positive mutation calls.

The interspecific F_1_ (group III) was a successful cross between the domesticated peach and its wild relative (*Prunus davidiana*) in 2005 (Supplemental Fig S6). The F_1_ individual was selfed in 2010 to derive F_2_ individuals. The pedigree to derive the F_1_ individual is described in Supplemental Fig S7. The F_2_ fruits were treated with low temperature storage for about one year and grown into small tress after successful germination (Supplemental Fig S6). Those F_2_ seeds were grown for about three years from 2012 to 2015 until sampling (Supplemental Fig S6). The parent tree was also sampled in 2015 with an age of about 10 years old. Leaves for the F_1_ parent tree and four of its ancestors as well as 30 of its progeny (Supplemental Table S1) were collected from an arbitrary branch of each individual. Note that the age of the F1 parental trees in crosses I and III are approximately the same, while that of cross II is much older. Should somatic mutations be causing a major difference between crosses I and III, the effect should be even more profound in cross II.

All of the F_1_ and F_2_ samples from group I and III were preserved in the National Fruit Tree Germplasm Repository, Zhengzhou Fruit Research Institute, Chinese Academy of Agricultural Sciences, China (Supplemental Table S1).

## b. Sequencing and alignment

Fresh leaves were collected from each plant, and stored at -80°C. DNA was extracted using CTAB method [1]. For two samples GZTH-5 and GZTH-8, the DNA was directly extracted from the seed after careful removal of the seed coat. All samples were sequenced using 150bp paired-end Illumina Hiseq4000 platform at BGI, with a library insert size of 350bp. Each sample was sequenced to at least 40×. Raw reads were cleaned by removing adaptors and low quality reads, ensuring over 95% of the clean data have a base quality ≥ 20 (e.g. Q20 ≥ 95%).

The high-quality whole-genome shotgun assembly of peach cv. Lovell was used as the reference genome [2]. We downloaded the latest version (Peach v2.0) of genome sequences from Genome Database for Rosaceae (GDR, <https://www.rosaceae.org/species/>prunus _persica/genome_v2.0.a1), this version contains several improvements of chromosome-scale assembly. All cleaned reads were mapped to the reference genome using BWA-mem 0.7.10-r789 [3] with option “-M”. The mapping results for each sample were sorted and stored in bam files. All bam files were processed with Picard tools MarkDuplicates version 1.114 to mark PCR duplicates, followed by local realignments around putative indel loci using RealignerTargetCreator and IndelRealigner in GATK package version 3.3.0 [4].

## c. Variant calling and marker identification

Initial variants for each sample were called using GATK HaplotypeCaller (HC). The HC was run in GVCF mode for each sample with default parameters, followed by combined genotyping across all samples within the same group. By default, HC requires a minimum mapping quality of 20 to generate confident calls. For mutation detection, raw variant calls were directly analysed without further filtering, as more pre-filtering steps would lead to a higher false negative rate.

For recombination analysis, markers with low confidence could hamper the identification of true recombinant blocks, therefore it is important to exclude false variant calls as thoroughly as possible. To generate a high confidence variant set, we only use bi-allelic variant loci with 1) quality ≥ 50; 2) a depth no less than 10 and not exceeding 80; 3) more than half of samples contain informative calls in each group. To reduce the genotyping errors, we also required a reference allelic ratio of 0~5% or 95%~100% to be considered as a confident homozygote, while 30%~70% was required to make a confident heterozygous call. A confident marker was thus identified where the F_1_ samples were present in a confident heterozygous status. This allele-balance filter is efficient for removing genotyping errors due to sequencing errors or possible contaminates, as those errors were most likely at a low frequency. However, mapping errors due to highly similar paralogous sequences could also result in pseudo-heterozygosity. To minimize these errors, we remove those markers residing in large structural variant (SV) regions of F_1_ samples compared to the reference genome in each group. The SVs were detected by combining three different algorithms: a read-depth approach (CNVnator) [5], a split-read approach (Pindel) [6] and from the analysis of discordant pairs (Breakdancer) [7]. CNVnator (version 0.3) was run with a bin size of 100bp, which predicts large deletions and duplications. Pindel (version 0.2.5b6) was run with default options. Results were collected for large deletions (≥ 100bp), inversions, and translocations. Deletion, duplication and inversion results were also collected from Breakdancer (version 1.1.2) with default settings. We generated a union set of results collected from all three approaches without further filtering. SVs with a size smaller than 100kbp were directly used. We also include 200bp flanking regions of all inversion events. For SVs larger than 100kbp, we use the 400bp flanking regions around each predicted SV breakpoint.

## *d. De novo* mutation identification

The candidate mutations raised in progeny were identified by searching for mutation alleles present in a single progeny only and not in the parent or other progeny of the same parent. Considering the number of F_2_ individuals sampled (10~30), it is unlikely for a mutation present in the parent tree to be passed only to a single progeny [8]. Thus this largely eliminates somatic mutations that arise early in the life of the parent tree, before the birth of those progeny. Branch specific somatic mutations may nonetheless be recovered, as will somatic mutations occurring early in the life of the progeny. We use a pipeline previously described [8] with slight modifications. The approach has a negligible false positive discovery rate and a circa 10% false negative rate [8]. We modified the detection pipeline in order to minimize any possible false negatives due to variant callers. We add another caller, UnifiedGenotyper (UG), also from the GATK package, to generate the initial variant sets. The UG was running with parameters “-glm BOTH -rf BadCigar -rf MappingQuality -mmq 20”, which requires a minimum mapping quality of 20. The following processing steps were then applied for both variant sets from HC and UG.

Genotyping errors in non-mutated samples could cause a failure to detect a true mutation with the same genotype called. To address this, we started from the rare variants with a frequency less than three in each group as the initial candidates. For all SNP candidates in each sample, we counted the covered reads for all present alleles in each strand using VarScan (version 2.3.6) readcounts [9]; for indel candidates we regenerated those indel calls by running HC in joint-calling model, from which a more accurate allele depth was obtained for each sample (present in AD field in generated VCF file). By direct comparing the reads covered upon each sample we purged genotyping errors and were able to efficiently remove false positives under the premise that reads from sequencing or mapping artefacts were less likely to shown only in a single sample.

Candidates mutations were detected by requiring 1) at least 5 reads with both forward and reverse strands in the focal sample (e.g. the sample carries a different allele from all other samples); 2) the parental samples should contain informative calls as a background, and no more than 5 “missing” data calls in other F_2_ samples (a high “missing” rate in each group is also a sign of low variant quality); 3) no evidence that the same mutationally-derived allele is present in either parental samples or other F_2_ progeny. All processed loci failing previous criteria were softmasked instead of direct hard filtering, and only loci passing all criteria were marked as “PASS”. We also masked loci with a clustering status (defined as more than three base substitutions within 10bp or more than two indels within 20bp) as those loci are most likely owing to contamination rather than true mutations.

Afterwards, all “PASS” candidates were manually investigated. The integrative genomics viewer (IGV) [10] was applied to review the mapping states across all samples within the candidate loci. We also extracted all aligned reads for each candidate locus from each sample and realigned those reads to the reference sequence with ClustalW2 [11] to get a more accurate alignment, and then manually inspect each alignment in combination with IGV. Candidate loci resulted from spurious mapping artefacts or possible contamination (detected by blast search in NCBI Nucleotide collection database using the aligned reads) were further discarded. Masked loci failing previous criteria were randomly sampled and also manually reviewed to make sure no true mutation was filtered out.

The final mutation results were obtained by combining all passed candidates from both UG and HC set. Most mutations were detected by both variant callers used. The consistency rate is higher for point mutations (213 of 240, 88.8%) than for indels (32 of 46, 69.6%). The HaplotypeCaller (HC) performs better in indel detection owing to a local re-assembly algorithm, 11 indel mutations were only called from HC while 3 were only called from UnifiedGenotyper (UG). For base substitution, UG missed 16 calls which could be detected by HC, while HC lost 11 calls predicted by UG. Although by fine-tuning parameters of each caller or following filtering procedures, most mutation calls could be recovered in another caller, as no prior knowledge of mutations was known before most studies, incorporating multiple algorithms can reduce the bias in mutation detection [12]. Our approach was also based on a direct parallel comparison among a large sample panel, as all the cohort samples share the same possibility of being an artefact at the same site, regardless of the regional context, which frees the assumption of less reliable results in hard-to-detect regions (e.g. low-complex regions, etc.). A softmasking strategy was also effective in controlling the false negatives (FN) and helps in adjusting the filtering criteria to obtain the best possible results. The whole detection pipeline (start from the raw variant sets) as well as accompanying scripts are available at <https://github.com/wl13/BioPipelines/tree/master/Mutation_Detection>.

## e. Sanger validation of mutation calls

We designed PCR primers for 101 randomly selected point mutations and 25 indel mutations, followed by Sanger sequencing to confirm those mutation calls. For each mutation locus, the F_2_ sample where this mutation was called, the F_1_ generation parental sample, and at least one additional F_2_ sample not supposed to carry the mutation were sequenced. Only mutation alleles verified in the called samples and not present in neither parental samples nor other F_2_ samples were considered as confirmed. Mutation loci failing to give valid results due to PCR difficulties or poor sequencing results were considered as undetermined.

**f. Estimation of mutation rate**

The per generation per site mutation rate was calculated by dividing the average number of called mutations by twice the accessible haploid reference genome size. The accessible reference genome size (i.e. callable sites) was estimated using a simulation approach described in Keightley et al, 2015 [13]. In outline, the empirical read-depth distribution for each group was sampled from F_2_s within sites with a confident heterozygous genotype in F_1_ parent (maker sites used in recombination analysis). The synthetic mutations were generated from all F_2_s using a custom PERL script sim_mutation_reads.pl ([https://github.com/wl13/ BioPipelines/](https://github.com/wl13/%20BioPipelines/)tree/master/Mutation_Simulation) with options “--random-size 1000 --samtools ‘-F 3844’ --exclude scaffold”. The samtools flag ignores those unmapped or supplementary reads that were not informative. A total of 1,000 synthetic mutations were generated on eight chromosomes for each group. These edited reads were then processed with the same pipelines to detect those synthetic mutations. The fraction of callable simulated mutated sites was used to estimate the fraction of callable sites in the genome for each group. Uncallable sites include sites that could not be called by any callers (e.g. due to low mapping quality), sites with a low-depth (<5) or strand bias (only have forward or reverse strands) in the mutated sample, and sites not informative in parental samples or other F_2_s. Among all callable sites, a total of 15 simulated sites failed to be recovered. Seven sites were due to high sequencing errors in other samples (>4 samples) or putative contamination in another sample, which happened to have an allele identical to the synthetic allele. Another 10 sites were found to reside in homopolymer or tandem repeat regions which also have indels nearby. These latter sites were actually captured as indel mutations and could be recovered through manual investigation. The overall false negative rate within callable sites was thus very low (<1%).

To estimate confidence intervals of the estimated mutation rate we assume the observed number of mutations is a poisson variable. We then apply the Poisson.test function in R to estimate confidence intervals, with confidence intervals set to 0.95.

**g. Estimation of heterozygosity**

For F_1_ samples in each group, the genome heterozygosity was estimated as the rate of heterozygous SNPs among all callable sites. This was done by genotyping each F_1_ sample including non-variant sites using GenotypeGVCFs “--includeNonVariantSites” option. For a confident heterozygous SNP, we require a minimum depth of 10 and a maximum depth of 80. We also calculated the reference allelic ratio, defined as proportion of reference-allelic reads to the total covered reads. Only SNPs with a reference allelic ratio between 30% to 70% were considered as a confident heterozygous call, while allelic ratios below 5% or above 95% were considered as a confident homozygous call. The same criteria were applied to all non-variant sites. The overall heterozygosity was estimated as number of heterozygous SNPs / (number of heterozygous SNPs + number of homozygous sites).

## h. Detection of crossover (CO) events

For interspecific F_2_ samples, we first genotyped each marker as *P. persica*-homozygous, *P. davidiana*-homozygous or heterozygous, by comparing against these parents. The markers were then clustered using a ‘seeding and extension’ approach to form the original inherited blocks. First, fragments with 25 consecutive markers of the same genotype and a length over 10kbp were chosen as a seed; adjacent seeds with same genotype were then merged into larger fragments (blocks) until all adjacent fragments were of different genotypes. Each block was further extended to the furthest marker of the same genotype where the overall proportion of this genotype started to decline. This algorithm has been implemented in the script “vcf_process.pl” and is available from https://github.com/wl13/BioScripts. Finally, all boundaries of blocks were manually inspected and revised. The final CO events were detected where the block’s genotype was switched, regions within those switched markers were identified as CO break regions.

For intraspecific *P. persica* group, it is difficult to first genotype each marker as neither of the parental individuals were available. Thus we only genotyped those markers as homozygous or heterozygous at first, and formed the blocks using the same clustering method mentioned above. This rests on the assumption of their being only a negligible chance for two crossover events to be observed in a very narrow region (i.e. within two adjacent markers) from a single F_2_ genome. This is reasonable as the two haplotypes of the same F_2_ genome came from independent meiotic processes. Once the initial blocks were formed, the F_1_ and other F_2_ chromosomes could then be phased according to those homozygous blocks (Supplemental Fig S1A). For each chromosome, we picked out a sample in which only a homozygous genotype was observed. As the selected sample consists of two identical haplotypes (defined as “Haplotype1”), the F_1_ chromosome as well as other F_2_ chromosomes could thus be phased through comparison to this haplotype (Supplemental Fig S1B). This process also relaxed the previous assumption and was robust to possible phasing errors (Supplemental Fig S1C). The final phased blocks were used to detect CO events as described before.

In order to make sure the stringent filtering steps did not remove many true variants and lead to an underestimation of CO events, we also identified inherited blocks and CO events before each filtering step was implemented. Through comparison of the CO events identified in those intermediate steps with the final results, we identified those filtered CO events that were always shared among many different individuals, which was not likely to happen in the randomly sampled F_2_ samples. Manual inspection of those regions also confirmed the non-proper mapping status and artefactual clustering of markers (standard error of distances between each two adjacent markers > 100) in those regions.

The *P. mira* F_1_ individual was estimated to have a slightly higher heterozygosity (0.0029) than *P. persica* F_1_ cross (0.0027), however, the mapping results of *P.* *mira* group were largely subjected to the genome rearrangements observed between *P. mira* and *P. persica*. Given a rough estimation, about half of the covered regions were associated with abnormal depth, non-proper insert size or orientation, which was even higher than estimated for *P. davidiana*. The large scale genomic rearrangement between *P. mira* and *P. persica* made the results less reliable as regards the CO results for *P. mira* group, therefore we did not include these results in the current study.

**i. Statistical analysis**

Statistics and correlation tests were performed in R [14]. Brunner-Munzel test was implemented in R package “lawstat”. The trinucleotide content of point mutations was counted with the mutation at the start, center and end of the triplet, and the mutation rate per given trinucleotide triplet was then calculated. The genome-wide trinucleotide content as well as triplets within heterozygous or homozygous compartments were also counted from the first, second and third nucleotide of each sequence. For each compartment, the expected number of point mutations was derived from the observed triplet mutation rate.

The CO coldspot and hotspot regions were detected by first dividing the whole genome in non-overlapping 500kbp windows. Midpoints of CO breaks were used as the location of CO events and were counted for each window. Windows with similar CO numbers were merged. All windows after merging were tested using a Monte Carlo process, with 10,000 randomizations of shuffling all CO events across whole genome to derive the *P* values. Regions with observed CO events significantly deviated (*P* < 0.05) from the expectation of randomizations were defined as hotspot regions (more than expectation) or coldspot regions (less than expectation), respectively.

To test whether the CO rate was correlated with the mutation rate, we binned the genome into 500kbp, 1Mbp, 2Mbp and 5Mbp domains. CO events and mutations were collected from both intraspecific *P. persica* group and interspecific group. Bins overlapping peri-centromeric regions were discarded due to recombination suppression in those regions. The relationship was tested using Spearman’s rank correlation.

To further test whether the CO rate was correlated with the intraspecific population diversity, 70 *P. persica* individuals were collected from published data [15]. All reads were mapped to the reference genome using BWA-backtrack algorithms [16], followed by marking of PCR duplicates (i.e. likely PCR amplification artefacts) and realignment processes as described before. Both variants and non-variant sites were called with HC in GVCF mode. Variant sites with more than half missing alleles or with a non-reference allele frequency < 7 (e.g. 5% of all 70 diploid individuals) were excluded to reduce false positive calls.

The population diversity was calculated as the average pairwise differences among all possible pairs. The pairwise difference was defined as the per site nucleotide difference between each of the two compared individuals, e.g. 1 would be counted for a difference between two different homozygous genotypes while 0.5 would be counted for a difference between a homozygous genotype and a heterozygous genotype. The pairwise differences were obtained by first summing up all nucleotide differences in a window, then dividing by the number of informative sites (sites genotyped in both individuals) in the same window. For each pair, only windows with more than 50% informative sites were considered as an informative pair in this window. Windows with less than 1208 informative pairs (e.g. 50% of all total 2415 pairs) were discarded from the correlation test.

**Supplementary References**

1. Murray, M. G. & Thompson, W. F. 1980 Rapid isolation of high molecular weight plant DNA. *Nucleic Acids Res.* **8**, 4321–4325.

2. The International Peach Genome Initiative et al. 2013 The high-quality draft genome of peach (Prunus persica) identifies unique patterns of genetic diversity, domestication and genome evolution. *Nat. Genet.* **45**, 487–494. (doi:10.1038/ng.2586)

3. Li, H. 2013 Aligning sequence reads, clone sequences and assembly contigs with BWA-MEM. *ArXiv13033997 Q-Bio*

4. DePristo, M. A. et al. 2011 A framework for variation discovery and genotyping using next-generation DNA sequencing data. *Nat. Genet.* **43**, 491–498. (doi:10.1038/ng.806)

5. Abyzov, A., Urban, A. E., Snyder, M. & Gerstein, M. 2011 CNVnator: An approach to discover, genotype, and characterize typical and atypical CNVs from family and population genome sequencing. *Genome Res.* **21**, 974–984. (doi:10.1101/gr.114876.110)

6. Ye, K., Schulz, M. H., Long, Q., Apweiler, R. & Ning, Z. 2009 Pindel: a pattern growth approach to detect break points of large deletions and medium sized insertions from paired-end short reads. *Bioinformatics* **25**, 2865–2871. (doi:10.1093/bioinformatics/btp394)

7. Chen, K. et al. 2009 BreakDancer: an algorithm for high-resolution mapping of genomic structural variation. *Nat. Methods* **6**, 677–681. (doi:10.1038/nmeth.1363)

8. Yang, S., Wang, L., Huang, J., Zhang, X., Yuan, Y., Chen, J.-Q., Hurst, L. D. & Tian, D. 2015 Parent-progeny sequencing indicates higher mutation rates in heterozygotes. *Nature* **523**, 463–467. (doi:10.1038/nature14649)

9. Koboldt, D. C. et al. 2012 VarScan 2: Somatic mutation and copy number alteration discovery in cancer by exome sequencing. *Genome Res.* **22**, 568–576. (doi:10.1101/gr.129684.111)

10. Thorvaldsdóttir, H., Robinson, J. T. & Mesirov, J. P. 2013 Integrative Genomics Viewer (IGV): high-performance genomics data visualization and exploration. *Brief. Bioinform.* **14**, 178–192. (doi:10.1093/bib/bbs017)

11. Larkin, M. A. et al. 2007 Clustal W and Clustal X version 2.0. *Bioinformatics* **23**, 2947–2948. (doi:10.1093/bioinformatics/btm404)

12. Alioto, T. S. et al. 2015 A comprehensive assessment of somatic mutation detection in cancer using whole-genome sequencing. *Nat. Commun.* **6**, 10001. (doi:10.1038/ncomms10001)

13. Keightley, P. D., Pinharanda, A., Ness, R. W., Simpson, F., Dasmahapatra, K. K., Mallet, J., Davey, J. W. & Jiggins, C. D. 2015 Estimation of the Spontaneous Mutation Rate in Heliconius melpomene. *Mol. Biol. Evol.* **32**, 239–243. (doi:10.1093/molbev/msu302)

14. R Development Core Team 2013 R Development Core Team (2013). R: A language and environment for statistical computing. R Foundation for Statistical Computing, Vienna, Austria. ISBN 3-900051-07-0, URL http://www.R-project.org.

15. Cao, K. et al. 2014 Comparative population genomics reveals the domestication history of the peach, Prunus persica, and human influences on perennial fruit crops. *Genome Biol.* **15**. (doi:10.1186/s13059-014-0415-1)

16. Li, H. & Durbin, R. 2009 Fast and accurate short read alignment with Burrows-Wheeler transform. *Bioinformatics* **25**, 1754–1760. (doi:10.1093/bioinformatics/btp324)
